# Differential gene expression in gall midge susceptible rice genotypes revealed by suppressive subtraction hybridization (SSH) cDNA libraries and microarray analysis

**DOI:** 10.1186/1939-8433-5-8

**Published:** 2012-04-03

**Authors:** Nidhi Rawat, Chiruvuri Naga Neeraja, Suresh Nair, Jagadish S Bentur

**Affiliations:** 1 Directorate of Rice Research, Rajendranagar, Hyderabad, 500 030 AP India; 2 International Centre for Genetic Engineering and Biotechnology, Aruna Asaf Ali Marg, New Delhi, 110067 India

**Keywords:** Compatible interaction, Susceptibility, Gall midge biotypes, Real-time PCR, Metabolic pathways

## Abstract

**Background:**

A major pest of rice, the Asian rice gall midge (*Orseolia oryzae* Wood-Mason), causes significant yield losses in the rice growing regions throughout Asia. Feeding by the larvae induces susceptible plants to produce nutritive tissue to support growth and development. In order to identify molecular signatures during compatible interactions, genome wide transcriptional profiling was performed using SSH library and microarray technology.

**Results:**

Results revealed up-regulation of genes related to primary metabolism, nutrient relocation, cell organization and DNA synthesis. Concomitantly, defense, secondary metabolism and signaling genes were suppressed. Further, real-time PCR validation of a selected set of 20 genes, in three susceptible rice varieties (TN1, Kavya and Suraksha) during the interaction with the respective virulent gall midge biotypes, also revealed variation in gene expression in Kavya as compared to TN1 and Suraksha.

**Conclusions:**

These studies showed that virulent insects induced the plants to step up metabolism and transport nutrients to their feeding site and suppressed defense responses. But Kavya rice mounted an elevated defense response during early hours of virulent gall midge infestation, which was over-powered later, resulting in host plant susceptibility.

**Electronic supplementary material:**

The online version of this article (doi:10.1186/1939-8433-5-8) contains supplementary material, which is available to authorized users.

## Background

Plant galls are defined as aberrant plant cells, tissues or organs formed as a result of stimulation by various parasites ranging from fungi and bacteria to insects and mites (Harris et al. 2003). Insects that induce plants to form galls are reported from diverse orders such as Diptera (gall midges), Hymenoptera (gall wasps), Homoptera (gall-forming aphids), Hemiptera (gall-forming psyllids) and Thysanopetra (thrips) (Saltzmann et al. 2008). The gall is the cumulative expression of a suite of adaptations in the host plant for accommodating herbivorous insects (Raman et al. 2011). Gall-inducing insects have profound effects on their hosts. These insects live within the plant tissues and induce tumor-like growths that provide them with food, shelter, and protection from natural enemies (Raman et al. 2005). While, tumors induced by bacteria, viruses and fungi have amorphous growth, galls induced by insects have symmetrical structures and display novel patterns of differentiation which do not occur during normal morphogenesis of the organ (Meyer 1987).

Gall-forming insects are also known to manipulate their host plants and induce changes in source-sink relationships in a way that is beneficial to larval development. Since insects derive their nutrition from gall tissue, the gall becomes a sink for different nutrients and energy that is vital for the insect's growth (Raman 2003; Raman and Abrahamson. 1995). The majority of gall-inducing insects stimulate the host-plant tissue to develop into galls through their feeding action, whereas species of Hymenoptera trigger gall development via oviposition. Even vascular tissues may be modified by gall induction, ensuring a supply of nutrients and water for the inducing insect (Meyer 1969). These insects, through feeding or oviposition, cause differentiation of a special nutritive tissue that is rich in sugars, proteins, and lipids, as well as a range hydrolyzing enzymes (Raman 2003; Raman and Ananthakrishnan. 1983).

Gall formation by plants due to insect attack has been interpreted as the defense strategy by various authors. Cockerell (1890) suggested that natural selection has preserved and accumulated the gall-forming tendencies. Weis and Kapelinski (1984) opined that "the relationship is strictly parasitic as the plant receives no benefit, and may even suffer a loss in reproductive output". Major hypotheses on the adaptive significance of insect gall formation have been reviewed by Price et al. (1987); these being nonadaptive, plant protections, mutual benefit, nutrition, microenvironment, and enemy hypotheses. According to Price et al. (1987) the evolution of the galling habit has followed two pathways, one via mining of plant tissues and the other through the modification of plant growth as carried out by sedentary external herbivores. Plants with galls suffer severe setbacks in growth and reproduction, whereas insects reproduce effectively in galls and multiply rapidly. However, Mani and Raman (1994) suggested that gall formation is advantageous to the plants as it favors survival of the attacked organ by restricting the insect parasite in space and time. Subsequently, plants could have evolved better defense strategy targeting insect "kill" than "accommodate".

Infestations of susceptible rice varieties by the Asian rice gall midge (*Orseolia oryzae* Wood-Mason) cause an average annual yield loss of about US$80 million in India and US$500 million in Asia (Bentur et al. 2003). This insect is essentially a monsoon pest and causes damage wherever high humidity and moderate temperatures prevail (Bentur et al. 2003). Feeding of the insect on the meristematic tissues of the growing terminal or the auxiliary shoot apices of the rice plant produces leaf sheath galls called silver shoots. Galls generally occur during the tillering stage and occasionally during panicle initiation and flowering (Kalode and Viswanathan. 1976). Early gall midge infestation results in profuse tillering and stunting, but few tillers bear panicles. Gall formation results from the suppression of leaf primordial differentiation at the growth cone and the development of radial ridges from the innermost leaf primordium, followed by elongation of the leaf sheath (Perera and Fernando. 1969). The life cycle of the insect is completed within 19-23 d, under normal temperature and humidity. The length and the size of the gall vary with the age of the plant and the sex of the insect. This pest has been effectively managed through the development and cultivation of resistant rice varieties. Single dominant resistance (*R*) genes confer resistance against the rice gall midge in the plant. So far 11 *R* genes have been identified. However, gall midge populations rapidly adapt to (become virulent against) *R* gene conferred resistance by evolving new biotypes. Rice-gall midge interactions are characterized as incompatible when the rice plant detects an avirulent larva through an *R*-gene mediated gene-for-gene recognition event that prevents larval establishment and results in larval death (Bentur et al. 2003). Incompatible interaction is manifested by two different mechanisms of resistance i.e. hypersensitive (HR + type) and non-hypersensitive (HR- type). In contrast, during a compatible interaction the virulent larva infests the host plant and successfully establishishes feeding sites at the base of the plant (the crown). To date seven distinct biotypes have been characterized in India (Vijaya Lakshmi et al. 2006). Because of this co-evolution, the rice-gall midge system is a useful research model to gain insights into genetic, molecular and evolutionary aspects of plant-insect interactions.

To understand the molecular mechanisms of rice-gall midge compatible interaction, rice genotypes TN1 (carrying no *R* gene) and Kavya (carrying an ineffective *Gm1* gene) were challenged with gall midge biotypes GMB4 and GMB4M, respectively. Whole genome transcriptome analysis was carried out by Suppressive Subtraction Hybridization (SSH) library construction and microarray analysis to identify candidate genes specifically involved in this interaction. We have also included another rice genotype, Suraksha (carrying an ineffective resistance gene *Gm11*, against GMB4M) for real-time PCR validation. Gall midge resistance genes in Suraksha (*Gm11*) and Kavya (*Gm1*) confer resistance via different mechanisms (i.e. HR + and HR- type, respectively). The purpose of including the Suraksha genotype for real-time PCR validation was to check whether the susceptibility phenomenon is similar or different in Suraksha and Kavya genotypes, under the background of different resistance genes when challenged with gall midge biotype virulent to both rice genotypes Suraksha and Kavya. Expression of a selected set of 20 genes were monitored and validated through real-time PCR for specific induction during early and late stage of interaction in three rice genotypes. Our study revealed that the compatibility in different genotypes of rice and gall midge biotypes shares differential expression patterns during early hours of infestation. Further, during later hours these genotypes share common pathways such as up-regulation of primary metabolism and transport related genes.

## Results

### Characterization of gall midge induced ESTs from the TN1 SSH library

We began the investigation by comparing gene expression in un-infested (control) and infested TN1 plants using SSH. Subtractive hybridization was performed using five-times more driver (control) than tester (infested) cDNA. After subtraction, the remaining cDNA was cloned into the lambda-ZAP vector and a total of 1450 clones were obtained. PCR was then used to identify clones containing inserts (1248) and each of these was sequenced. Of these, 1248 PCR positive clones were sequenced and analyzed. After removing low quality sequences and short sequence reads, 552 ESTs were considered for sequence assembly to reveal 309 singletons and the remaining 243 sequences were assembled in 131 contigs [Additional file 1: Table S1, Rawat et al. (unpublished data)] using the MacVector program. These high quality sequences had an average read of 500 bps. All the unique ESTs were submitted to the EST database of GenBank http://www.ncbi.nlm.nih.gov/dbEST (accession no. HO188242-HO188793). The library served to elucidate transcriptional changes and subsequent differential responses in the rice variety TN1 triggered by infestation with the rice gall midge biotype 4 (GMB4). Based on homology search of BLASTX and BLASTN, among 440 non-redundant sequences of the TN1 library, 370 clones (84%) were homologous to known genes, 48 clones (11%) were hypothetical and 23 clones (5%) did not show any hits in the rice database.

### Characterization of gall midge induced ESTs from Kavya identified using microarrays

The compatible interaction in Kavya-GMB4M was studied using microarray analysis. Of 51,279 probe sets, contained in the Affymatrix Rice GeneChip, 50,382 produced detectable hybridization signals under our experimental conditions. The total number of probe sets for analysis was reduced to 24,150 transcripts by removing probe sets with ambiguous signals and those that were not called "present" in at least two replicates at one time interval. Among these transcripts, 1330 genes recorded at least two-fold changes in expression levels (either up or down) and a *p* value < 0.05 in paired *t* tests between un-infested control and GMB4M infested samples [Additional file 1: Table S1; Rawat et al. (unpublished data)]. Of these 1330 induced transcripts, 789 were up-regulated and 541 were down-regulated in comparison with the un-infested control Kavya.

### Comparative analysis of gall midge induced genes

We further analyzed the genes that appeared to be the major targets for differential regulation during the compatible interactions using MapMan software (Usadel et al. 2005) with the *Oryza sativa* mapping file. The data obtained from both approaches were screened for similarities and were categorized into thirteen differentially expressed groups (Additional file 1: Table S1) since both approaches cannot be compared because of different genotypes used in the studies.

#### Commonly induced genes

The SSH data provided only up-regulated genes while microarray data provided both up- and down-regulated genes. Commonly induced genes comprised much of the data of the up-regulated genes from both compatible interactions. Various genes from seven different groups such as primary metabolism, protein synthesis, transporters, cell wall metabolism, transcription factors, redox and development were commonly up-regulated in both studies.

For both genotypes, a large number of differentially expressed genes representing primary metabolism such as carbohydrate, lipid, protein, nitrogen and nucleotide metabolism were identified. Genes related to glycolysis such as fructose-bisphosphate aldolase and glyceraldehyde-3-phosphate dehydrogenase (Additional file 2: Figure S1 and Additional file 3: Figure S2), and those involved in the TCA cycle i.e. gene pyruvate dehydrogenase E1 component were further validated using real-time PCR. The genes involved in lipid biosynthesis, such as those coding omega-6 fatty acid desaturase and nonspecific lipid-transfer protein were also represented in both the interactions.

**Figure 1:**
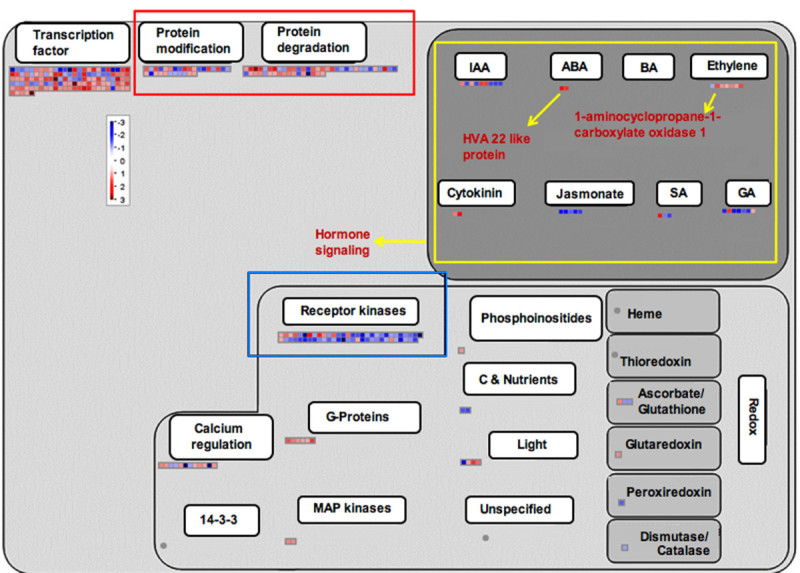
**MapMan-based visualization of the differentially expressed genes involved in 'regulation overview' in the microarray analysis of the rice variety Kavya after infestation with GMB4M**. Functional subBINs (small squares) shown in red or blue indicate their up-regulation or down-regulation, respectively. SubBINs shown by yellow, red and blue rectangle represent differentially expressed genes involved in hormone signaling; protein modification and degradation; and receptor kinase activity, respectively. Grey circles indicate the genes unchanged or changed by less than 2-fold. Colour key represents log_2_ scale.

**Figure 2:**
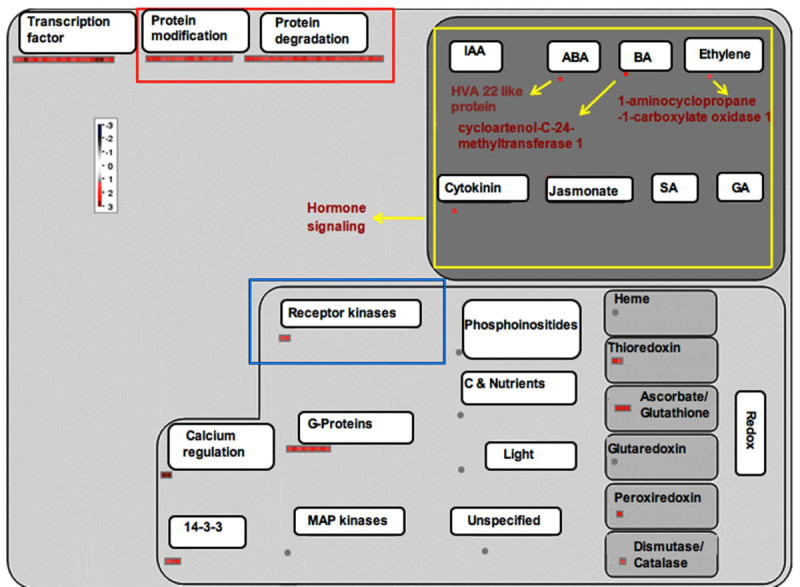
**MapMan-based visualization of the differentially expressed ESTs involved in 'regulation overview' in the SSH cDNA library of the rice variety TN1 after infestation with GMB4**. Functional subBINs (small squares) shown in red indicate their up-regulation. SubBINs shown by yellow, red and blue rectangle represent differentially expressed genes involved in hormone signaling; protein modification and degradation; and receptor kinase activity, respectively. Grey circles indicate the genes unchanged or changed by less than 2-fold. Colour key represents log_2_ scale.

In the present study, "protein synthesis and turnover" group comprises the maximum numbers of differentially expressed genes (105 genes in Kavya and 101 genes in TN1) which can be further classified into five subgroups (Figure 1 and 2). The first and second subgroups were comprised of ribosomal proteins and translation initiation factors, respectively. The 60S ribosomal protein L37, L30 and elongation factor Tu were induced in both genotypes. The third subgroup of post translational modification related genes such as protein phosphatase 2 C, chaperonin and mitogen-activated protein kinase kinase kinase (MAPKKK) were commonly enriched. The fourth subgroup consisted of protein targeting related genes such as importin alpha and mitochondrial-processing peptidases and these were commonly up-regulated. The fifth subgroup consisted of protein degradation related genes from both genotypes and included ubiquitin conjugating enzyme E2, zinc finger C_3_HC_4_ type family protein, F-box domain containing protein, and proteasome subunit alpha type 1.

Eight genes of the"transporters related" group coding for ATPases transporter, sugar transporter, phosphate transporter, ABC transporter and major intrinsic proteins such as aquaporin PIP and NIP were commonly induced in both the compatible interactions. Cell wall metabolism related genes involved in cell-wall loosening or in basic component synthesis exhibited higher expression levels during these interactions. Genes involved in cell-wall modification and loosening during growth include xyloglucan, β-expansin, pectinesterase and endo-1, 4-β glucanases. Genes, involved in the cell wall synthesis such as cellulose synthase, UDP-glucose 6-dehydrogenase and UDP-glucuronic acid decarboxylase were also up-regulated. Several cell wall proteins encoding genes including proline rich proteins were found in both the sets. Our studies revealed induction of various transcription factors in both the compatible interaction such as GRAS family of transcription factors named DELLA protein RGL1 and chitin-inducible gibberellin-responsive protein. Other commonly induced genes in both the studies were Homeobox transcription factor family OCL4 protein, MADS-box transcription factor 18, auxin response factor, AP2-binding protein, Zn finger proteins, bZIP transcription factor, NAC domain containing protein and PHD-finger family protein. Further, genes involved in oxidative burst such as peroxiredoxin, glutaredoxin and catalase and development related genes such as senescence-associated protein DH, nodulin-like protein, B12D protein were commonly up-regulated in both the studies.

#### Differentially induced genes

Genes related to group 6 such as DNA synthesis/chromatin remodeling and repair, cell division, phenylpropanoid pathway, defense, hormone signaling and cell signaling were differentially expressed in both the genotypes.

**Figure 3:**
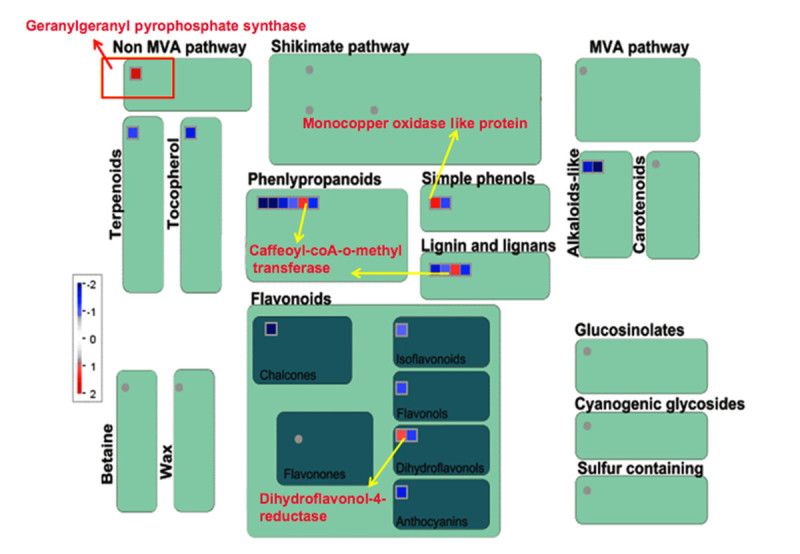
**MapMan-based visualization of the differentially expressed genes involved in 'secondary metabolism' in the microarray analysis of the rice variety Kavya after infestation with GMB4M**. Functional subBINs (small squares) shown in red or blue indicate their up-regulation or down-regulation, respectively. Differentially regulated genes are marked with red rectangle and arrows (yellow and red). Grey circles indicate the genes unchanged or changed by less than 2-fold. Colour key represents log_2_ scale.

**Figure 4:**
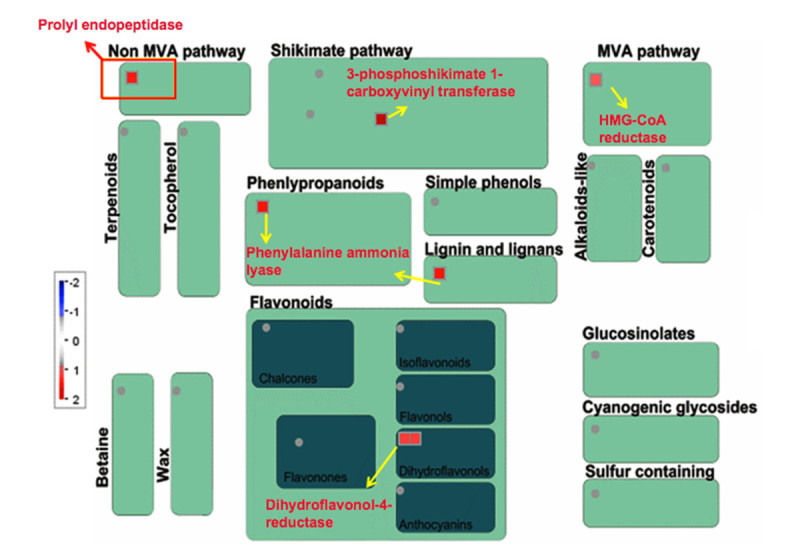
**MapMan-based visualization of the differentially expressed ESTs involved in 'secondary metabolism' in the SSH cDNA library of the rice variety TN1 after infestation with GMB4**. Functional subBINs (small squares) shown in red indicate their up-regulation. Differentially regulated genes are marked with red rectangle and arrows (yellow and red). Grey circles indicate the genes unchanged or changed by less than 2-fold. Colour key represents log_2_ scale.

Eighteen genes pertaining to DNA synthesis/chromatin remodeling were induced only in Kavya and these coded for the h/ACA ribonucleoprotein complex subunit, DNA primase, replication factor and DNA polymerase (Additional file 4: Figure S3). Four genes related to DNA repair, replication protein A, formamidopyrimidine-DNA glycosylase, DNA repair protein RAD51 and DNA binding protein were also up-regulated in Kavya (Additional file 4: Figure S3). Cell division related genes coding for meiotic recombination protein DMC1, ribosome recycling factor, mitotic spindle checkpoint protein MAD2 and CDC45-related protein were present only in Kavya (Additional file 4: Figure S3 and Additional file 5: Figure S4). Five genes related to secondary metabolism in TN1 and three genes in Kavya were up-regulated. However, 13 genes related to secondary metabolism were down-regulated in Kavya. A gene encoding dihydroflavonol -4- reductase was commonly up-regulated in both the studies (Figure 3 and 4). Non-mevolnate pathway of diterpene synthesis related gene gernylgernyl pyrophosphate synthase in Kavya (Figure 3) and prolyl endopeptidase in TN1 (Figure 4) were up-regulated. Phenylalanine ammonia lyase genes were up-regulated in TN1 data but were found to be down-regulated in Kavya. Genes encoding for terpene synthase, anthranilate N-benzoyltransferase, chalcone synthase, flavonol synthase and isoflavone reductase were among down-regulated genes present in Kavya (Figure 3). Genes of the "defense" related group i.e. those encoding for leucine rich repeat family protein (3 ESTs), ethylene response protein, serine/threonine-protein kinase SAPK9 and EDS1-like protein were found in the TN1 genotype. Genes specifically up-regulated in Kavya coded for dirigent-like protein, dehydrin family protein, lectin precursor, thionin precursor, NADPH oxidase, *atrbohF* and NBS-LRR disease resistance protein (Additional file 6: Figure S5 and Additional file 7: Figure S6). In the control several genes of defense related group such as pathogenesis related proteins, xylanase inhibitor protein, germin-like protein, chaperone protein dnaJ, thaumatin-like protein, wound inducive gene, endochitinase, NBARC and disease resistance protein RGA3 were down-regulated in Kavya (Additional file 6: Figure S5). Hormone signaling related cytokinin pathway and ethylene signaling pathways were up-regulated in Kavya while genes related to the jasmonic acid, gibberelin and salicylic acid pathways were down-regulated (Figure 1 and Additional file 6: Figure S5). A brassinosteroid signaling related gene named cycloartenol-C-24-methyltransferase was present only in TN1 (Figure 2 and Additional file 7: Figure S6). Only a few genes related to cell signaling were up-regulated in compatible interactions such as receptor kinases leucine rich repeat III and leucine rich repeat XI. But most of the cell signaling related genes including those coding for DUF26 receptor kinase, wall associated protein kinase, receptor kinases and sugar and nutrient signaling were down-regulated in Kavya.

#### Real-time PCR validation of selected genes

Real-time PCR analysis was conducted for a set of 20 shortlisted differentially expressed genes (Table 1) which were commonly (12 genes) or specifically (5 genes in TN1 and 3 genes in Kavya) induced in both the compatible interactions involving TN1-GMB4 and Kavya-GMB4M. To understand the 'susceptibility' phenomenon in rice-gall midge interaction, we also included a third independent set of compatible interactions involving Suraksha-GMB4M, for real-time PCR validation of the selected genes as described in the material and method.

**Table 1:** Primer sequence information for 20 selected genes chosen for real-time PCR assay

S.No	Gene Name	Locus ID	Primer sequences	Tm (°C)	Product size (bp)
1	geranylgeranyl pyrophosphate synthetase	loc_os02g44780	F: GACAGGTTCGGTGACAAGTATGG R: CGCATAGTCCACGAAGCTGTAC	60	63
2	cycloartenol-C-24-methyltransferase 1	loc_os07g10600	F: TGGATCCAAGCCGGTTTTC R: AATTGCACGCCCCACAGTA	59	59
3	outer mitochondrial membrane protein porin	loc_os03g10510	F: CCAAGTACAGTGCTGCGATAGG R: GCCAGAACGATTGCAGCAT	58	61
4	peptide transporter PTR2	loc_os05g33960	F: TACTACCTCGTCTGCTTCCACTTCT R: TCACACTCCTTCTCGTCATCGT	59	80
5	fructose-bisphosphate aldolase	loc_os01g67860	F: CCATCGCAGCTTTCCATTG R: TCCTGGCAGATGATGGCATA	60	65
6	glyceraldehyde-3-phosphate dehydrogenase	loc_os06g45590	F: CAAGGCTGGAATTGGCTTAAGTT R: GTAGCCCCACTCGTTGTCGTA	58	70
7	pyruvate dehydrogenase E1 component	loc_os06g13720	F: ATCGAAAAGCCCCGCATACT R: TGACTGCAAGAACATCCATACCA	59	80
8	catalase-1	loc_os03g03910	F: TCAACCGCAACATCGACAAC R: TCCCCGGCACGATGATC	62	75
9	DELLA protein RGL1	loc_os03g48450	F: ACTGTGCCACGGGATGATG R: CTTCCCGGCCAAATAGATCTC	60	61
10	NADH oxidase	loc_os01g53294	F: GAAAGAGGAAAAGCCGAAAAGG R: CCCACCGGATTACCGAAAC	59	69
11	*Atrboh F*	loc_os05g38980	F: TGGGTCTCCAACACTTACGAAA R: TCGTTGTCGTCTGGCTGAATT	58	65
12	NBS-LRR	loc_os02g55550	F: ACTATGCTCCCGGTTCCCTAA R:ATCGGTCAGTGAAGAGCAGTGA	58	130
13	NAC domain containing protein	loc_os08g10080	F: AAGGAGGACTGGGTGCTATGC R: TCTTCAGATGATGGGCTTGGA	58	71
14	TCTP	loc_os11g43900	F: GATGGCGGCTTGGTGTTT R: CAGCCCATGAGAGAAGTAAAGGA	60	75
15	Chitin inducible gibberallin	loc_os07g36170	F: CCAATCCAACGTCTAGGTGCTT R: TTTGTGCCAGAGTTTCCATGTCTA	58	68
16	Substrate Transporter	loc_os06g02370	F: GCAGCTTCCTCGGCATCAT R: CTTCAGCGCCCCGATGT	59	62
17	ABC transporter	loc_os04g38570	F: CGAATGCAATGGAGAGGAAGAC R: ACCGCATATAACCCAGTTCCAA	57	67
18	CBS domain containing protein	loc_os03g52690	F: TATCGGGTCACATGGCAGTCT	58	70
			R: AATAGATGGTTTTGGAGCACTGATG		
19	Lipase	loc_os09g22450	F: AAGCAAAGCATGATGCAAGAGAT R: GCTCAGTTCAAGGCCTCCAA	58	111
20	OsMADS18 - MADS-box family gene	loc_os07g41370	F:CGGGAGGAGCAAAATGGA R:GAGCCGTCACTGGTGTTGGT	60	58

#### Genes induced at 24 h after GMB4M infestation

**Figure 5:**
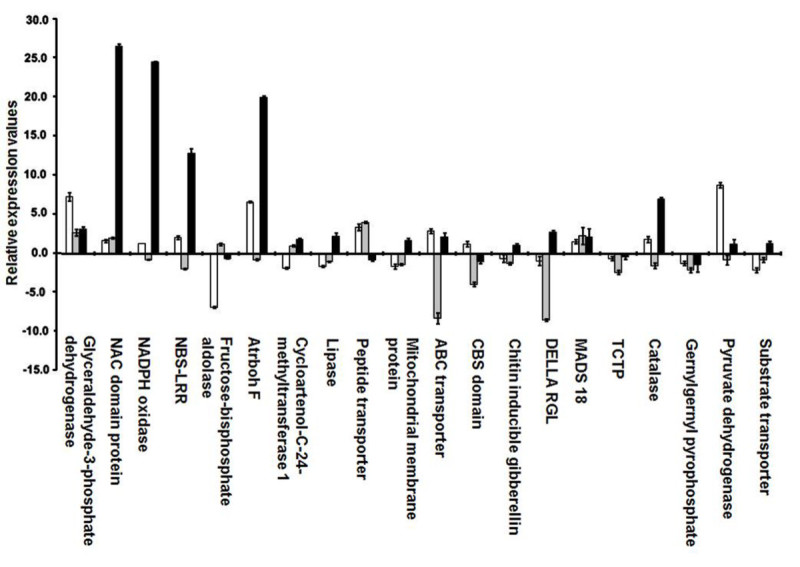
**Relative expression profiles of 20 shortlisted genes in TN1, Suraksha and Kavya at 24 h after GMB4M infestation**. Error bars represent Mean ± S.D. White, grey and black bars represent compatible interactions of TN1-GMB4M, Suraksha-GMB4M and Kavya-GMB4M, respectively.

The three compatible interactions examined showed different expression patterns at 24 h after gall midge infestation (Figure 5). Of the 20 genes validated, five defense and stress related genes viz., NADPH oxidase (24.4 fold), *atrbohF* (20.0 fold), NBS-LRR (12.8 fold), NAC domain containing protein (26.4 fold), and catalase (7.0 fold) were up-regulated with higher fold values in Kavya when compared with the respective values in TN1 and Suraksha (Figure 5). All these five genes were up-regulated in the TN1-GMB4M interaction also, but with lower values (~2.0 fold), except *atrbohF* (6.0 fold). However, these five genes were found to be down-regulated in Suraksha. NAC domain containing protein showed just 2.0 fold up-regulation. Another five genes viz., DELLA-RGL, cycloartenol-C-24-methyltransferase, lipase, outer mitochondrial membrane protein porin and substrate transporter were specifically up-regulated (~2 fold) only in Kavya and found to be down-regulated in TN1 and Suraksha. Early induction of these genes in Kavya suggested that despite being ineffective, the *Gm1* gene mounted a resistance response to the virulent attack of GMB4M and signified the involvement of these genes in the compatible interaction Kavya-GMB4M.

Two genes coding for glycolysis pathway glyceraldehyde-3-phosphate dehydrogenase and pyruvate dehydrogenase E1 component were up-regulated (7.2 fold and 8.5 fold, respectively) in TN1 but not in Kavya and Suraksha. The gene coding for peptide transporter PTR2 was up-regulated in TN1 and Suraksha (~3.5 fold) but down-regulated in Kavya. Six genes viz., CBS domain protein, Chitin inducible gibberellin, TCTP, geranylgeranyl pyrophosphate synthetase, fructose-bisphosphate aldolase and substrate transporter were down-regulated in all three genotypes. However, one gene coding for MADS18 was up-regulated of all three genotypes at 24 h after gall midge attack.

#### Genes induced at 120 h after GMB4M infestation

**Figure 6:**
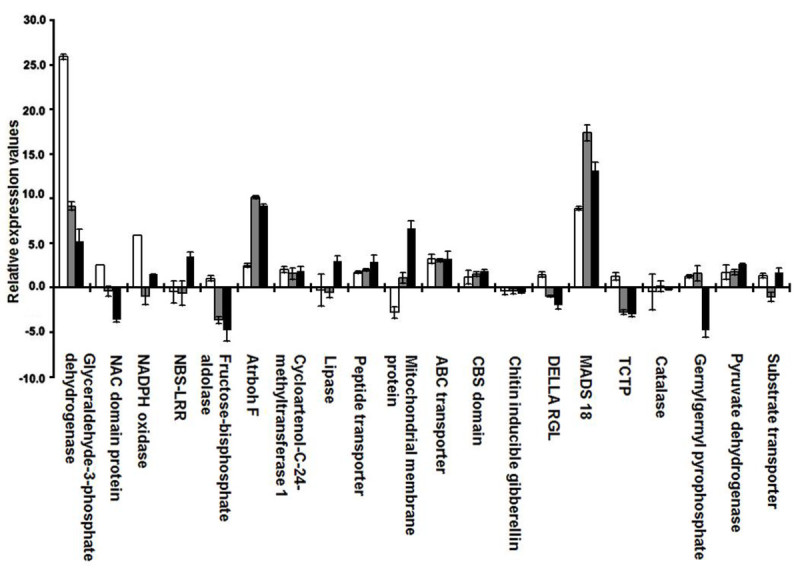
**Relative expression profiles of 20 shortlisted genes in TN1, Suraksha and Kavya at 120 h after GMB4M infestation**. Error bars represent Mean ± S.D. White, grey and black bars represent compatible interactions of TN1-GMB4M, Suraksha-GMB4M and Kavya-GMB4M, respectively.

The expression patterns of most of the 20 genes were uniform and in the same direction in all three genotypes at 120 h after GMB4M infestation (Figure 6). Seven genes coding for glyceraldehyde-3-phosphate dehydrogenase, *atrbohF*, cycloartenol-C-24-methyltransferase 1, peptide transporter PTR2, ABC transporter, MADS 18 and pyruvate dehydrogenase E1 component were significantly up-regulated with similar values in the three genotypes. However, at 24 h of these seven genes, glyceraldehyde-3-phosphate dehydrogenase and pyruvate dehydrogenase E1 component in TN1 and, *atrbohF* and cycloartenol-C-24-methyltransferase 1 in Kavya were up-regulated with comparatively higher fold values, respectively than in Suraksha. Two genes coding for chitin inducible gibberallin and *atrbohF* were down-regulated in all three interactions. At 24 h, catalase was up-regulated only in Kavya but chitin inducible gibberallin was down-regulated in all the three genotypes.

Six genes viz., fructose-bisphosphate aldolase, CBS domain containing protein, DELLA RGL, TCTP, geranylgeranyl pyrophosphate synthetase and substrate transporter did not showed any significant change in all the three genotypes. At 24 h, fructose-bisphosphate aldolase and CBS domain containing protein were down-regulated and DELLA RGL was up-regulated specifically in Kavya, but TCTP, geranylgeranyl pyrophosphate synthetase and substrate transporter were down-regulated in all the genotypes. The remaining five genes showed specific up-regulation in one of the genotypes including two genes in TN1 (NAC domain containing protein and NADPH oxidase) and three genes in Kavya (NBS-LRR, lipase and outer mitochondrial membrane protein porin). No gene was specifically up-regulated in Suraksha.

## Discussion

The majority of published studies to date have focused on plant transcript profiles of compatible interactions with phloem-feeding insects. These plant transcript profiling studies revealed that photosynthesis-related genes were down-regulated in response to phloem-feeding by insects such as aphids in sorghum (Zhu-Salzman et al. 2004), brown planthopper in rice (Yuan et al. 2005), and by whitefly in *Arabidopsis* (Kempema et al. 2007). Recent reports on phloem feeding insects (PFI) suggest that these insect induce cell wall modifications, reduce photosynthetic activity, manipulate source-sink relations, and modify secondary metabolism in their hosts (Thompson and Goggin 2006). Unlike other herbivores, PFIs up-regulated genes involved in nitrogen assimilation (Zhu-Salzman et al. 2004). In particular, aphids induced genes encoding enzymes required for synthesis of tryptophan and certain other amino acids (Moran et al. 2002). Sugar depletion at PFI feeding sites created localized metabolic sinks by inducing genes involved in carbon assimilation and mobilization (Moran et al. 2002; Zhu-Salzman et al. 2004). For example, green peach aphid feeding on celery petiole up-regulated genes implicated in remobilizing mannitol reserves (Divol et al. 2005). A common feature among the transcript profiling studies of sap sucking insects has been the identification of genes encoding proteins that alter cell wall structure. Genes encoding cell wall-modifying enzymes such as xyloglucan endotransglycosylase (XTH) and pectin methyl esterases were modulated in infested plants against phloem feeding insects (Moran et al. 2002). Phloem feeding insects can significantly reduce photosynthetic rates in their host plants. Transcript profiling has revealed that PFI infestation down-regulates expression of photosynthesis-related genes, such as those required for Rubisco synthesis (Yuan et al. 2005). Sap sacking insects cause a reduction in phenylpropanoid related transcripts. For example, in rice, brown planthopper feeding down-regulated several genes involved in phenylpropanoid biosynthesis and up-regulated a gene required for sesquiterpene synthesis (Cho et al. 2005). Hormone signaling also plays a crucial role in susceptibility as in wheat; SA induction was observed in incompatible but not in compatible interactions with the Russian wheat aphid (Mohase and van derWesthuizen 2002). A few isozymes of lipoxygenase involved in JA synthesis, were also induced by aphid feeding in sorghum (Zhu-Salzman et al. 2004). Studies on a rice-BPH compatible interaction showed decreases in nitrogen content, in photosynthetic activity and in sucrose content leading to lower production of nutrient assimilate and the disruption of translocation of these assimilates in susceptible rice. In wheat-Hessian fly compatible interaction studies, soluble nutrients such as free sugars were depleted (Zhu et al. 2008). A decrease in content of free sugars was compensated by the transport of photoassimilates from other parts of the plants to the feeding site. Our study corroborates these reports. In the present study, up-regulation of the soluble starch synthase gene in both sets of compatible interactions was observed. A common phenomenon in both compatible interactions was up-regulation of genes involved in primary metabolism, nutrient metabolism, transporters, and DNA synthesis and down-regulation of defense and secondary metabolism that suppresses the plant resistance pathway against the pest.

### Stimulation of primary metabolism

For many plant parasites, attack on the susceptible host plant is associated with the creation of a zone of "metabolic habitat modification" (Goethals et al. 2001). In this zone, the parasite experiences a selective advantage because of enhanced host nutrition and reduced defense. Developmental changes and stress responses are often correlated with or result in adjustment in various metabolic pathways. Primary metabolism plays a crucial role in susceptibility/resistance in the plant after pest recognition.

The present study provided evidence for increased and improved food quality through up-regulation of genes involved in primary metabolism. Genes of glycolysis, TCA cycle and carbohydrate metabolism were up-regulated in both the compatible interactions (Additional file 2: Figure S1 and Additional file 3: Figure S2). A gene coding for glyceraldehyde-3-phosphate dehydrogenase, a gene involved in the glycoytic pathway, for instance, was induced in both sets and a gradual increase in the transcript levels at two time points as shown by real-time PCR further supported the generalization that primary metabolism was indeed geared up.

### Enhanced nutrient metabolism

Earlier studies of the rice-gall midge interaction reported increased amino acid concentration within the host favoring gall midge multiplication (Regupathy and Subramanian. 1972) and probably indicated the role of amino acids in the susceptibility of plant. In the present study, genes encoding for amino acid synthesis and metabolism were down-regulated in Kavya but were up-regulated in TN1. The free amino acid content increases in the wheat plant following Hessian fly infestation to provide support for insect survival since N-compounds, such as amino acids, may provide better nutrition to insects. The dramatic C/N ratio shift in plants during compatible interactions might be a necessity for the insect growth and development and, therefore, for host plant susceptibility (Harris et al. 2006). The coordinated activation of the amino acid synthesis pathways, glycolysis, and the TCA cycle noted in the present study may have resulted in a shift in the C/N ratio.

### Induction of transporters and DNA synthesis related genes

Our study also showed induction of a large number of transcripts related to transporters in both compatible interactions. Evidence for increased nutrient flow came from the strong up-regulation of genes that encode various transporters i.e. peptide transporter PTR2, outer mitochondrial membrane protein porin, ABC transporter, amino acid transporter and substrate transporters. An increased nutrient flow has also been reported in the wheat-Hessian fly compatible interaction (Liu et al. 2007). The up-regulation of these transporters may imply nutrient flow from other parts of the plant to the midge feeding site. The increase in transporters may also hint at the activity of the insect in neutralizing plant toxins by effluxing them out of the area.

Genes related to DNA structure, synthesis and repair, and cell organization such as cell division and cell cycling were significantly up-regulated in Kavya (Figure 1). This is in contrast to the wheat-Hessian fly compatible interaction (Zhu et al. 2008). Involvement of these genes may indicate an altered course of morphogenesis to produce vegetative growth. Up-regulation of genes for cell wall metabolism, specially cell wall degradation and modification, may facilitate gall formation and loss of cell wall due to the action of cell wall degradation enzymes and may facilitate the establishment of tissue for feeding.

### Suppression of defense and secondary metabolism

In both compatible interactions a large number of genes encoding for abiotic stress responses such as DnaJ protein, chaperon, and heat shock proteins were commonly up-regulated. The greater stress response during compatible interactions may be a reflection of greater stress, not threat, on the susceptible plant by the virulent larva (Anderson and Harris. 2006; Harris et al. 2006; O'Donnell et al. 2003; Woodward and Bartel. 2005). However, down-regulation of the genes related to biotic defense pathways including NB-ARC, PR genes, xylanase inhibitors and disease resistance proteins and genes related to secondary metabolism, specially phenylpropanoid pathway genes such as PAL and anthranilate N-benzoyltransferase protein, suggest that the virulent larvae effectively suppressed the plant's defenses.

### Hormonal changes

Plant hormone signaling pathways are not isolated but rather interconnected with complex regulatory networks involving various defense signaling pathways and developmental processes (Bari and Jones. 2009). We observed up-regulation of genes involved in synthesis of auxin, abscissic acid (ABA), cytokinin (CK) and down-regulation of genes involved in synthesis of salicylic acid (SA), jasmonic acid (JA) and gibberellins (GA) in both the compatible interactions (Figure 1 and 2).

Several studies have shown that successful pathogen infection results in imbalances in auxin levels as well as changes in the expression of genes involved in auxin signaling. For example, infection with *Pst DC3000* results in increased IAA levels in *Arabidopsis* (O'Donnell et al. 2003). To regulate plant growth and development, auxin can induce the expression of three groups of genes: The Aux/IAA family, the GH3 family and the small auxin-up RNA (SAUR) family (Woodward and Bartel. 2005). We observed the induction of the OsIAA7-Auxin-responsive Aux/IAA gene family (os.7355.1.s1_at), the indole-3-acetic acid-amido synthetase GH3.2 (os.12501.1.s1_at) and the OsSAUR39 - Auxin-responsive SAUR gene family (os.39652.1.s1_at) in Kavya.

In general, ABA is involved in the negative regulation of plant defenses against various biotrophic and necrotrophic pathogens. Exogenous application of ABA enhances susceptibility of various plant species to bacterial and fungal pathogens. For example, application of ABA enhances the susceptibility of *Arabidopsis* plants to *Pst* (De Torres-Zabala et al. 2007). We observed the up-regulation of abscisic acid related genes encoding for HAV22-like protein in both interactions. Up-regulation of the ABA pathway genes may be involved in susceptibility against gall midge attack. Cytokinins (CK) are plant hormones involved in diverse processes of cell differentiation and growth (Bari and Jones. 2009). We found up-regulation of genes related to the cytokinin pathway in both studies. Genes involved in cytokinin homeostasis (cytokinin synthases and cytokinin oxidases/dehydrogenases) are strongly down-regulated in the resistance interaction of *P. brassicae* infected *Arabidopsis* (Siemens et al. 2006).

Three phytohormones--SA, JA and ET, are known to play major roles in regulating plant defense responses against various pathogens, pests and abiotic stresses such as wounding and exposure to ozone (Balbi and Devoto 2008). Several studies indicate that JA- and ET-signaling often operate synergistically to activate the expression of some defense related genes after pathogen inoculation (Glazebrook 2005). Down-regulation of these three hormones in both interactions signifies the suppression of defense signaling in the rice plant upon virulent gall midge infestation. Gibberellins (GA) promote plant growth by stimulating degradation of negative regulators of growth called DELLA proteins. We observed up-regulation of the GRAS family of genes in both compatible interactions.

Further, real-time PCR validation for the 20 selected genes at two time points provided a better insight into different rice-gall midge compatible interactions. Real-time PCR results indicated that the three genotypes displayed different responses at 24 h, but a common response at 120 h, after gall midge infestation. Defense and stress related genes were up-regulated more in Kavya than in Suraksha and TN1 at 24 h including the NAC domain containing gene, NADPH oxidase, *atrbohF*, NBS-LRR and catalase. The NAC genes are involved in host response to pathogen infection and other stress factors. NADPH oxidase and *atrbohF* are the genes responsible for respiratory burst oxidase to generate reactive oxygen species (ROS) during stress and NBS-LRR is a widely implicated *R* gene motif involved in plant defense against pathogens and insects. Catalase is a ROS scavenging enzyme that detoxifies the ROS generated in plants during pathogen infection. Induction of these genes specifically in Kavya and further real-time PCR validation suggested that despite being ineffective against GMB4M, the *Gm1* gene mounted an active resistance response during early hours of infestation. However, that was transient in nature and declined during the course of the virulent infestation. However down-regulation of these genes in Suraksha suggests that the *Gm1* gene in Kavya has a comparatively stronger defense response than the *Gm11* gene in Suraksha during the early period of insect infestation.

A few genes were specifically down-regulated in Suraksha. During the early hours of infestation, these included DELLA RGL, CBS domain protein and ABC transporter. Our results indicate the involvement of passive resistance in Suraksha that might help the virulent insect to survive. Up-regulation of primary metabolism related genes such as genes from the glycolytic pathway during early hours of infestation in TN1 suggests that the gall midge infestation quickly reprogrammed the plant's metabolic machinery towards accommodation of the pest. Similar gene expression patterns in all three genotypes at 120 h indicate that the counter defense imposed by the virulent insect takes over the 'pest-detection system' of the plant after a certain period resulting in its susceptibility.

## Conclusions

The present study describes the molecular events and cellular reprogramming in compatible interactions of two different rice verities after infestation by the virulent gall midge biotypes. The common phenomenon revealed by both the studies was up-regulation of primary metabolism, nutrient metabolism and transporters, and DNA synthesis aiding survival of the virulent larva. Further, down-regulation of defense and secondary metabolism suppressed the plant resistance against the pest. However, different host genotypes elicited diverse responses during the early stage of infestation.

## Methods

### Plant material and gall midge biotypes

Three rice (*Oryza sativa* L.) genotypes "TN1", "Suraksha" and "Kavya" and two gall midge biotypes, gall midge biotype 4 (GMB4) and gall midge biotype 4 M (GMB4M) maintained in a greenhouse at the Directorate of Rice Research, Hyderabad, India (Himabindu 2009) were used in the present study. While TN1 has no resistance gene, Kavya has the *Gm1* gene and Suraksha has *Gm11* both of which are ineffective against the two gall midge biotypes used in this study. Seeds of all three genotypes were sown in six rows 5 cm apart with 10-15 plants per row in three plastic trays (60 × 30 × 30 cm) filled to 8 cm deep with puddle soil. One plastic tray with 15 day old seedlings of test genotypes was exposed to GMB4 and a similar tray was exposed to GMB4M with 25 female and 10 male adults per tray. A third un-infested tray of test plants was used as control. After 48 h of insect release, these trays were transferred to a high humidity chamber (> 90% RH) where they were maintained for two days for egg incubation and maggot establishment. Plants were regularly observed for egg hatching and sampled at five different time intervals after egg hatching (Rawat et al. 2010).

### Experimental treatments and RNA extraction

Tissue samples of infested (TN1-GMB4, TN1-GMB4M, Suraksha-GMB4M and Kavya- GMB4M) and uninfested plants (uninfested TN1, Suraksha and Kavya) were collected at different time intervals (24 h, 48 h, 96 h and 120 h after egg hatching), separately. For RNA extraction, the basal part of the plants, above the root, were dissected in RNALater (Ambion, USA) to remove living and dead maggots, and the meristematic tissue from the feeding site was preserved. For each time interval, total RNA was extracted from the meristematic stem tissues using TRIzol (Invitrogen, USA) according to manufacturer instructions. Equal amounts of RNA were pooled from each time interval sampling for infested and control test samples, separately. The pooled RNA from TN1 and Kavya were used for the construction of a SSH cDNA library and microarray hybridization. However the RNA from Suraksha was used for real-time PCR validation. The RNA samples from all the three genotypes were further purified with DNAase (Qiagen Inc., Valencia, CA, USA) through an RNeasy Plant Mini kit according to the manufacturer's instructions (Qiagen Inc., Valencia, CA, USA). Purified DNAase-treated RNA was analyzed on a ND-1000 spectrophotometer (Nanodrop Technologies, Wilmington, DE) to determine the quantity and purity of the sample. RNA samples were used only if the 260/280 ratio was between 1.9 and 2.2.

### Isolation of induced ESTs during the TN1-GMB4 interaction using SSH cDNA library

Equal amounts of total RNA from all the time intervals were pooled separately for both the control (700 μg) and infested (350 μg) TN1 plants. mRNA was isolated from the total RNA by magnetic separation after annealing to 5'-biotinylated oligo dT primer and subsequently immobilized on streptavidin-linked paramagnetic beads (Mishra et al. 2005). First strand cDNA was synthesized using Superscript III reverse transcriptase (Invitrogen, USA) from control plants and was subsequently used as driver (uninfested TN1). The driver and the tester (infested TN1-GMB4) were mixed at a 5:1 ratio. Subtraction Hybridization was carried out to normalize the sequence abundance according to Mishra et al. (2005) to enrich the differentially expressed clones involved in the gall midge response.

Double strand cDNA was synthesized using the sequence abundant normalized and enriched differentially up-regulated, subtracted mRNA. The double stranded cDNA was uni-directionally ligated to the lambda-ZAP vector, in-vitro packaged and allowed to infect XL1- Blue MRF *E.coli* cells according to the manufacturer's instructions using a UNI-ZAP XR cDNA library construction kit (Stratagene, La Jola, California).

### Screening of the SSH library, sequencing and data analysis

The individual recombinant plaques from the primary cDNA library were manually transferred into 96-well format flat bottom storage plates containing 200 ml of SM buffer (100 mM NaCl, 8 mM MgSO4, 7H_2_O, 50 mMTris-HCl pH 7.0, 0.04% gelatin) supplemented with 5% DMSO and 0.5% chloroform and stored temporarily at 48°C until verification of the size of cDNA insert by PCR. The cDNA clones were PCR amplified with 100 ng of universal, M13 forward and reverse primers. Recombinant phages having insert sizes of 200 bp were re-arrayed into new storage plates. PCR was performed using 1 μl of phage suspension as template in 100 μl of PCR reaction volume (1XPCR buffer, 200 μM dNTPs and 5U Taq polymerase) for 30 cycles with following PCR conditions, 94°C for 1 min, 55°C for 1 min and 72°C for 2 min. The rearrayed (> 200 bp) PCR products were purified using the StrataPrep 96 PCR purification kit (Stratagene, USA) to remove un-incorporated primers and nucleotides. The purified PCR products were sent for sequencing (Macrogen, South Korea).

The sequences obtained were processed using the MacVector (MacVector Inc, USA, version 11.1.2) suite of programs to trim out vector sequences. The inbuilt Phred and Phrap program of MacVector was used and the sequences assembled into contigs and singletons. An homology search was performed using BLASTX and BLASTN http://www.ncbi.nlm.nih.gov/BLAST/ to search for homologous sequences in the non-redundant nucleotide and protein database. Functional classification of the ESTs was performed with the GO@EBI http://www.ebi.ac.uk/GO/. All the processed sequences have been submitted to the GenBank at NCBI and assigned accession numbers HO188242-HO188793.

### Isolation of induced transcripts during the kavya-GMB4M interaction using microarray analysis

RNA extraction, labeling, and hybridization for microarray were conducted using infested Kavya with GMB4M and uninfested plants as a control. Single-stranded and double-stranded cDNA was synthesized using the SuperScript Double-Stranded cDNA Synthesis Kit (Invitrogen Corp., Carlsbad, CA). Biotin labeling and cRNA fragmentation were carried out according to the Affymetrix GeneChip Expression Analysis Technical Manual http://media.affymetrix.com/support/downloads/manuals/expression_analysis_technical_manual.pdf.

### Statistical analysis of microarray data

The array data set was analyzed using the GeneChip Operating Software (GCOS 1.4). We used a default target intensity value (TGT) set at 500. The scaling factor for the arrays ranged from 3.1 to 8.5. The detection calls (present, absent, or marginal) for the probe sets were made by GCOS. CEL files generated by GCOS were imported into Avadis 4.3 (Strand Life Sciences, India) for further analysis. Normalization was performed using the robust multichip average (RMA) algorithm, and only gene expression levels with statistical significance (*p* < 0.05) above background levels were recorded as being "present". To determine the changes in gene expression between control and infested Kavya, we treated the 'uninfested' sample as a baseline control. The expression values were log 2 transformed after calculating the expression index. The signal log ratio is the change in the expression level of a transcript between the control and experimental samples, and log 2 (ratio) ≥ 1 (2-fold change) or log 2 (ratio) ≤ -1 (0.5-fold change) with *p* < 0.05 was used as cutoff. The analysis was performed using 'Limma' software from R-package http://www.r-project.org. Functional analyses of the differentially expressed genes were conducted with the MapMan software based on the Wilcoxon Rank Sum Test. All microarray data from the present investigation are available from NCBI GEO under the series entry GSE22538 http://www.ncbi.nlm.nih.gov/geo.

### Functional annotation and metabolic pathway analysis using MapMan software

Functional annotation and metabolic pathway analysis were performed using MapMan (Usadel et al. 2005). MapMan can be used to identify the functional categories associated with a set of sequences (e.g. differentially expressed) and thus find the metabolic pathways or other cellular functions up- or down-regulated in microarray experiments. The mapping file functionally classified all the genes of an Affymetrix rice chip into 36 major BINs (groups) and several subBINs. As Mapman software was used for microarray data analysis we have converted locus IDs of induced ESTs, obtained after the analysis of the SSH library, to probe IDs and compare the induced genes identified from both studies.

The functional classification in the mapping file (Osa_affy_150909) that structures the rice gene from an Affymetrix into distinct metabolic and cellular processes from the MapMan program was used. Differentially expressed rice genes were functionally annotated by performing BLAST alignment against the TIGR database. MapMan software was employed to show the differences in gene expression in different cellular and metabolic process. Ratios were expressed in a log_2_ scale for importing into the software and changes in expression were displayed via a false color code. The locus IDs of induced ESTs in the SSH library were converted into corresponding Probe IDs using http://www.ricearray.org to compare the SSH library data with microarray data using MapMan software.

### Quantitative real-time PCR for validation of differentially expressed genes

The real-time PCR was conducted for the 20 shortlisted genes identified from the SSH cDNA library and microarray analysis. For real-time PCR validation a third set of compatible interactions (Suraksha-GMB4M) was also included along with TN1-GMB4M and Kavya- GMB4M. These three genotypes vary with respect to the presence of gall midge resistance genes viz., TN1 (no *R* gene), Suraksha (carrying *Gm11 R* gene, conferring HR + type resistance) and Kavya (carrying *Gm1 R* gene, conferring HR- type resistance). GMB4M is virulent on all three genotypes. The real-time PCR was validated in three biological replicates for all the compatible interactions with two points (24 h and 120 h after GMB4M infestation) and compared with their respective control plants.

Real-time PCR was performed using the Applied Biosystems 7500 real-time PCR System with the SYBR green chemistry (Applied Biosystems, USA) according to the manufacturer's instructions. Gene-specific primers for the real-time PCR were designed using Primer Express Software (Applied Biosystems). The rice ubiquitin gene, *OsUBC* (accession no. AK059694) was used as the endogenous control. PCR reactions were carried out in a 10 μl reaction containing 2 μl of first strand cDNA, 1X PCR buffer, 125 μM dNTPs, 1.5 mM MgCl2, 0.2 μM primers and 1U Taq polymerase. The thermal profile used was: 94°C for 2 min; 35 cycles at 94°C for 20 s, annealing at 60°C for 20 s, 72°C for 30 s and final extension of 72°C for 5 min. Melt curve analysis was also performed after completion of PCR cycles to check specificity of the PCR amplification. To calculate the mean relative expression levels, cDNAs from three independent biological samples each in three technical replications were used. Relative transcription levels are presented graphically on the log scale.

## Electronic supplementary material

Additional file 1:**Table S1**. List of total up-regulated genes and description of the differentially expressed genes in both studies reported from thirteen different groups. (XLS 310 KB)

Additional file 2:**Figure S1**. MapMan-based visualization of the differentially expressed genes involved in 'glycolysis' in the microarray analysis of the rice variety Kavya after infestation with GMB4M. Functional subBINs (small squares) shown in red or blue indicate their up-regulation or down-regulation, respectively. Differentially regulated genes are marked with red arrows. Red rectangles represent commonly up-regulated genes in both the compatible interactions. Grey circles indicate the genes unchanged or changed by less than 2-fold. Colour key represents log_2_ scale. (TIFF 8 MB)

Additional file 3:**Figure S2**. MapMan-based visualization of the differentially expressed ESTs involved in 'glycolysis' in the SSH cDNA library of the rice variety TN1 after infestation with GMB4. Functional subBINs (small squares) shown in red indicate their up-regulation. Differentially regulated genes are marked with red arrows. Red rectangles represent commonly up-regulated genes in both the compatible interactions. Grey circles indicate the genes unchanged or changed by less than 2-fold. Colour key represents log_2_ scale. (TIFF 8 MB)

Additional file 4:**Figure S3**. MapMan-based visualization of the differentially expressed genes involved in 'cellular response' in the microarray analysis of the rice variety Kavya after infestation with GMB4M. Functional subBINs (small squares) shown in red or blue indicate their up-regulation or down-regulation, respectively. Red rectangle represents differentially expressed genes involved in cell division, cell cycle and development related pathways. Red arrow highlights up-regulated genes involved in cell division in Kavya-GMB4M interaction. Grey circles indicate the genes unchanged or changed by less than 2-fold. Colour key represents log_2_ scale. (TIFF 8 MB)

Additional file 5:**Figure S4**. MapMan-based visualization of the differentially expressed ESTs involved in 'cellular response' in the SSH cDNA library of the rice variety TN1 after infestation with GMB4. Functional subBINs (small squares) shown in red indicate their up-regulation. Red rectangle represents differentially expressed genes involved in cell division, cell cycle and development related pathways. Grey circles indicate the genes unchanged or changed by less than 2-fold. Colour key represents log_2_ scale. (TIFF 8 MB)

Additional file 6:**Figure S5**. MapMan-based visualization of the differentially expressed genes involved in 'biotic stresses' in the microarray analysis of the rice variety Kavya after infestation with GMB4M. Functional subBINs (small squares) shown in red or blue indicate their up-regulation or down-regulation, respectively. Differentially regulated genes are marked with red arrows. Purple rectangles represent differentially expressed genes involved in hormone signaling; proteolysis; cell wall synthesis, modification and degradation. Grey circles indicate the genes unchanged or changed by less than 2-fold. Colour key represents log_2_ scale. (TIFF 7 MB)

Additional file 7:**Figure S6**. MapMan-based visualization of the differentially expressed ESTs involved in 'biotic stresses' in the SSH cDNA library of the rice variety TN1 after infestation with GMB4. Functional subBINs (small squares) shown in red indicate their up-regulation. Differentially regulated genes are marked with red arrows. Purple rectangles represent differentially expressed genes involved in hormone signaling; cell wall synthesis, modification and degradation; proteolysis and transcription factors. Grey circles indicate the genes unchanged or changed by less than 2-fold. Colour key represents log_2_ scale. (TIFF 8 MB)

Below are the links to the authors’ original submitted files for images.

Authors’ original file for figure 1

Authors’ original file for figure 2

Authors’ original file for figure 3

Authors’ original file for figure 4

Authors’ original file for figure 5

Authors’ original file for figure 6

Authors’ original file for figure 7

Authors’ original file for figure 8

Authors’ original file for figure 9

Authors’ original file for figure 10

Authors’ original file for figure 11

Authors’ original file for figure 12
