# Supplementary figures and images for: Differential gene expression in gall midge susceptible rice genotypes revealed by suppressive subtraction hybridization (SSH) cDNA libraries and microarray analysis
Source: Rice (N Y). 2012 Apr 3;5:8. doi: 10.1186/1939-8433-5-8 (PMC5520839; doi:10.1186/1939-8433-5-8)

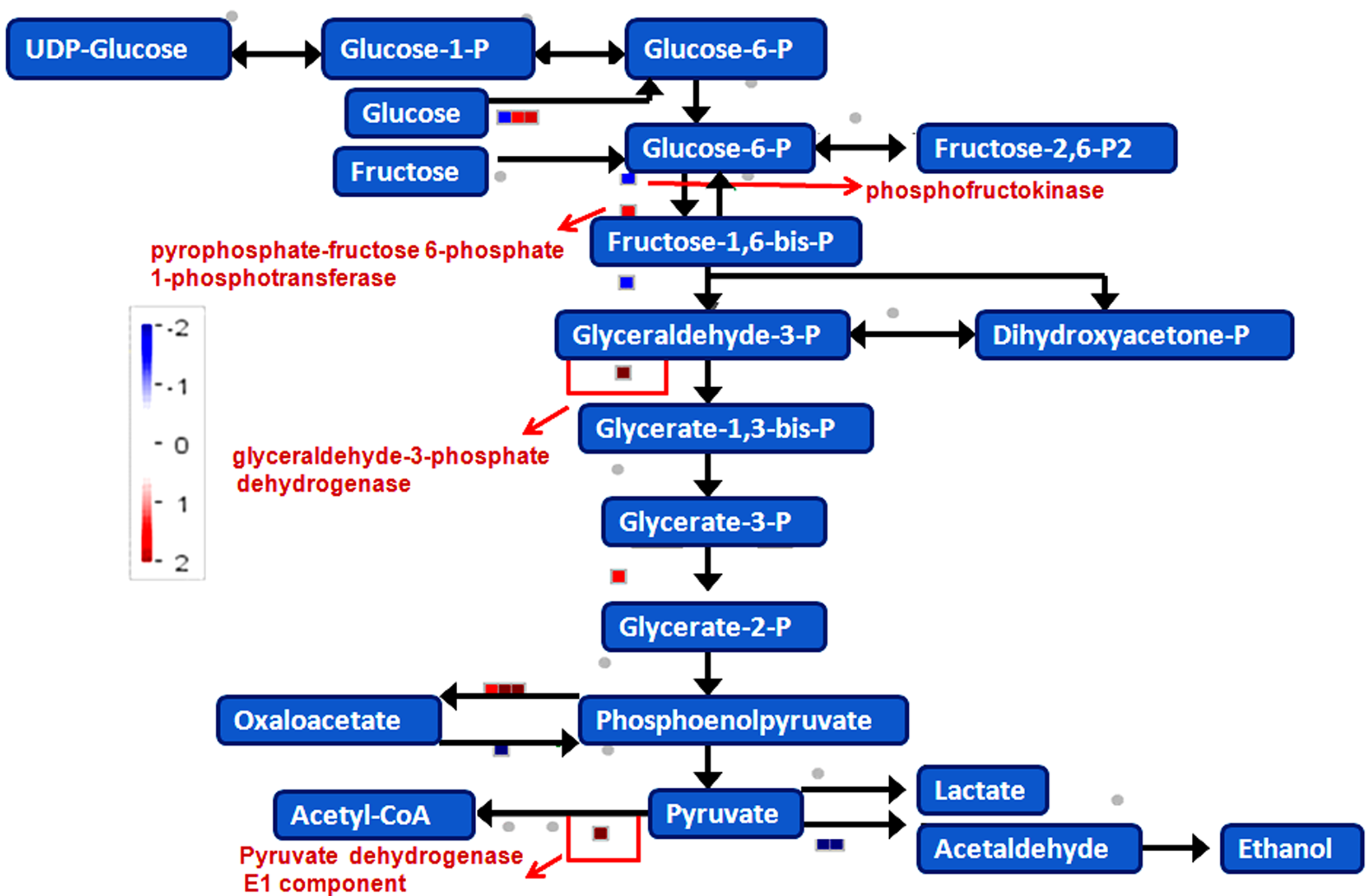

Supplement: Supplementary file 2 — Additional file 2:Figure S1. MapMan-based visualization of the differentially expressed genes involved in 'glycolysis' in the microarray analysis of the rice variety Kavya after infestation with GMB4M. Functional subBINs (small squares) shown in red or blue indicate their up-regulation or down-regulation, respectively. Differentially regulated genes are marked with red arrows. Red rectangles represent commonly up-regulated genes in both the compatible interactions. Grey circles indicate the genes unchanged or changed by less than 2-fold. Colour key represents log2 scale. (TIFF 8 MB) [file 12284_2011_8_MOESM2_ESM.TIFF]

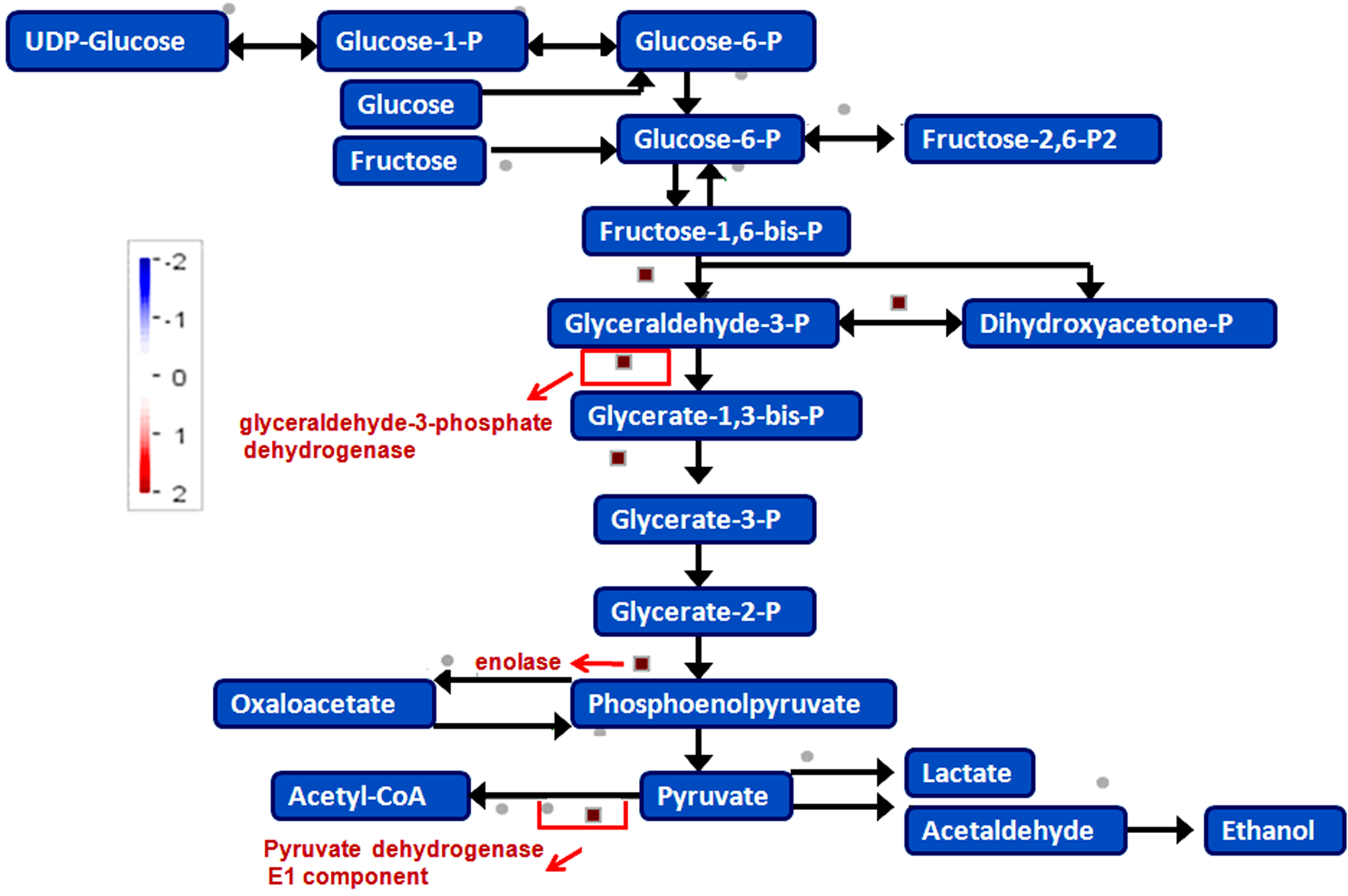

Supplement: Supplementary file 3 — Additional file 3:Figure S2. MapMan-based visualization of the differentially expressed ESTs involved in 'glycolysis' in the SSH cDNA library of the rice variety TN1 after infestation with GMB4. Functional subBINs (small squares) shown in red indicate their up-regulation. Differentially regulated genes are marked with red arrows. Red rectangles represent commonly up-regulated genes in both the compatible interactions. Grey circles indicate the genes unchanged or changed by less than 2-fold. Colour key represents log2 scale. (TIFF 8 MB) [file 12284_2011_8_MOESM3_ESM.TIFF]

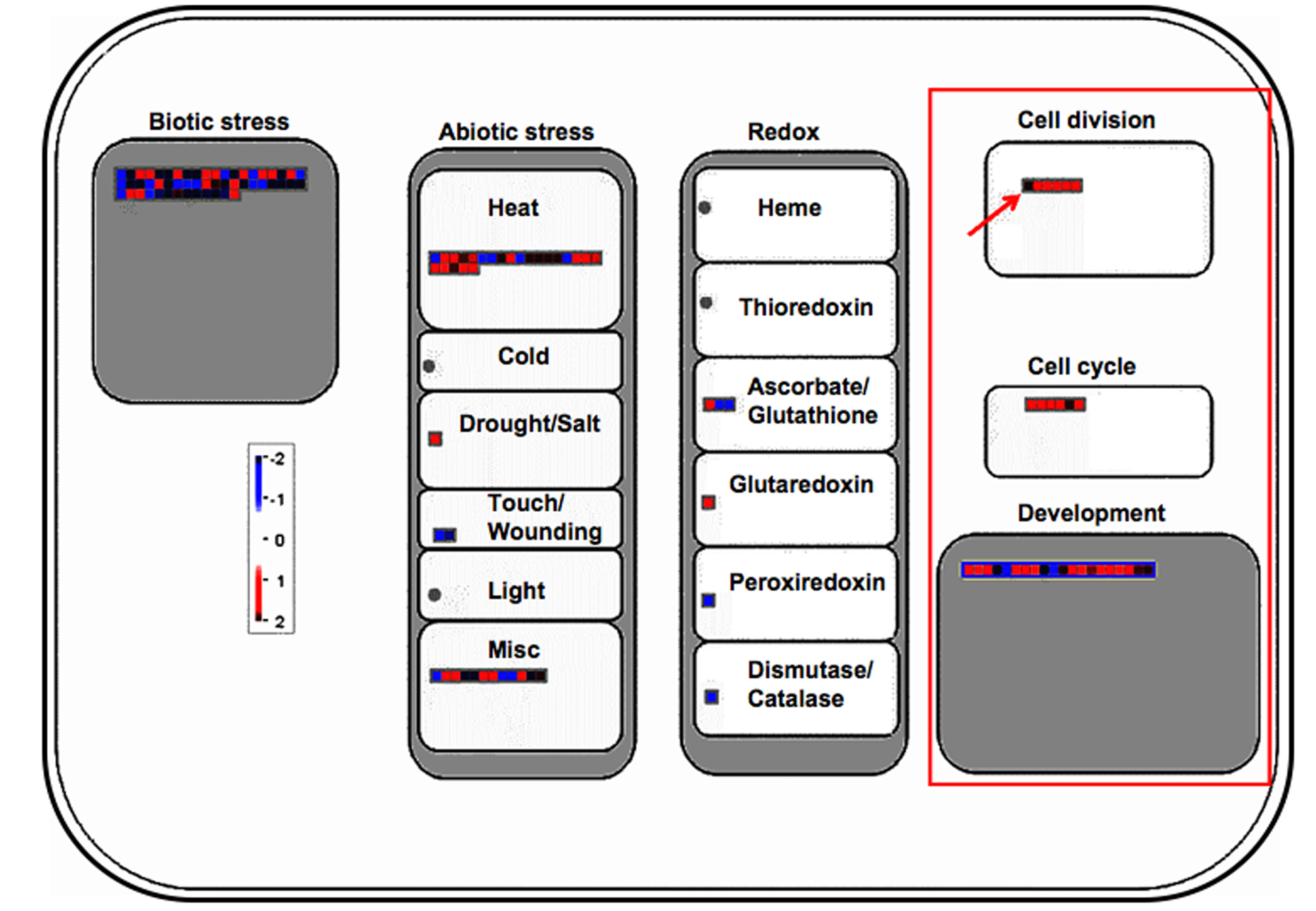

Supplement: Supplementary file 4 — Additional file 4:Figure S3. MapMan-based visualization of the differentially expressed genes involved in 'cellular response' in the microarray analysis of the rice variety Kavya after infestation with GMB4M. Functional subBINs (small squares) shown in red or blue indicate their up-regulation or down-regulation, respectively. Red rectangle represents differentially expressed genes involved in cell division, cell cycle and development related pathways. Red arrow highlights up-regulated genes involved in cell division in Kavya-GMB4M interaction. Grey circles indicate the genes unchanged or changed by less than 2-fold. Colour key represents log2 scale. (TIFF 8 MB) [file 12284_2011_8_MOESM4_ESM.TIFF]

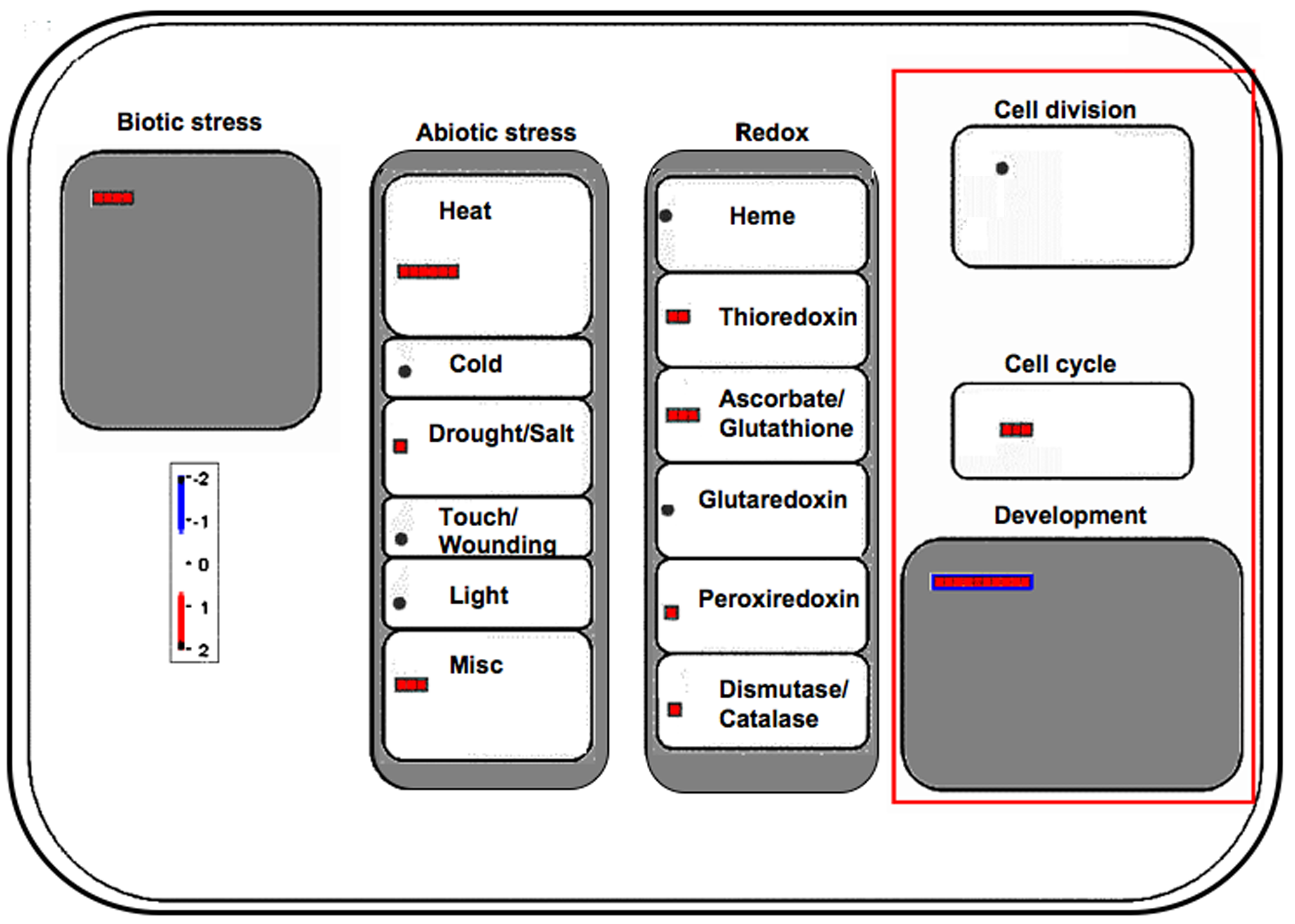

Supplement: Supplementary file 5 — Additional file 5:Figure S4. MapMan-based visualization of the differentially expressed ESTs involved in 'cellular response' in the SSH cDNA library of the rice variety TN1 after infestation with GMB4. Functional subBINs (small squares) shown in red indicate their up-regulation. Red rectangle represents differentially expressed genes involved in cell division, cell cycle and development related pathways. Grey circles indicate the genes unchanged or changed by less than 2-fold. Colour key represents log2 scale. (TIFF 8 MB) [file 12284_2011_8_MOESM5_ESM.TIFF]

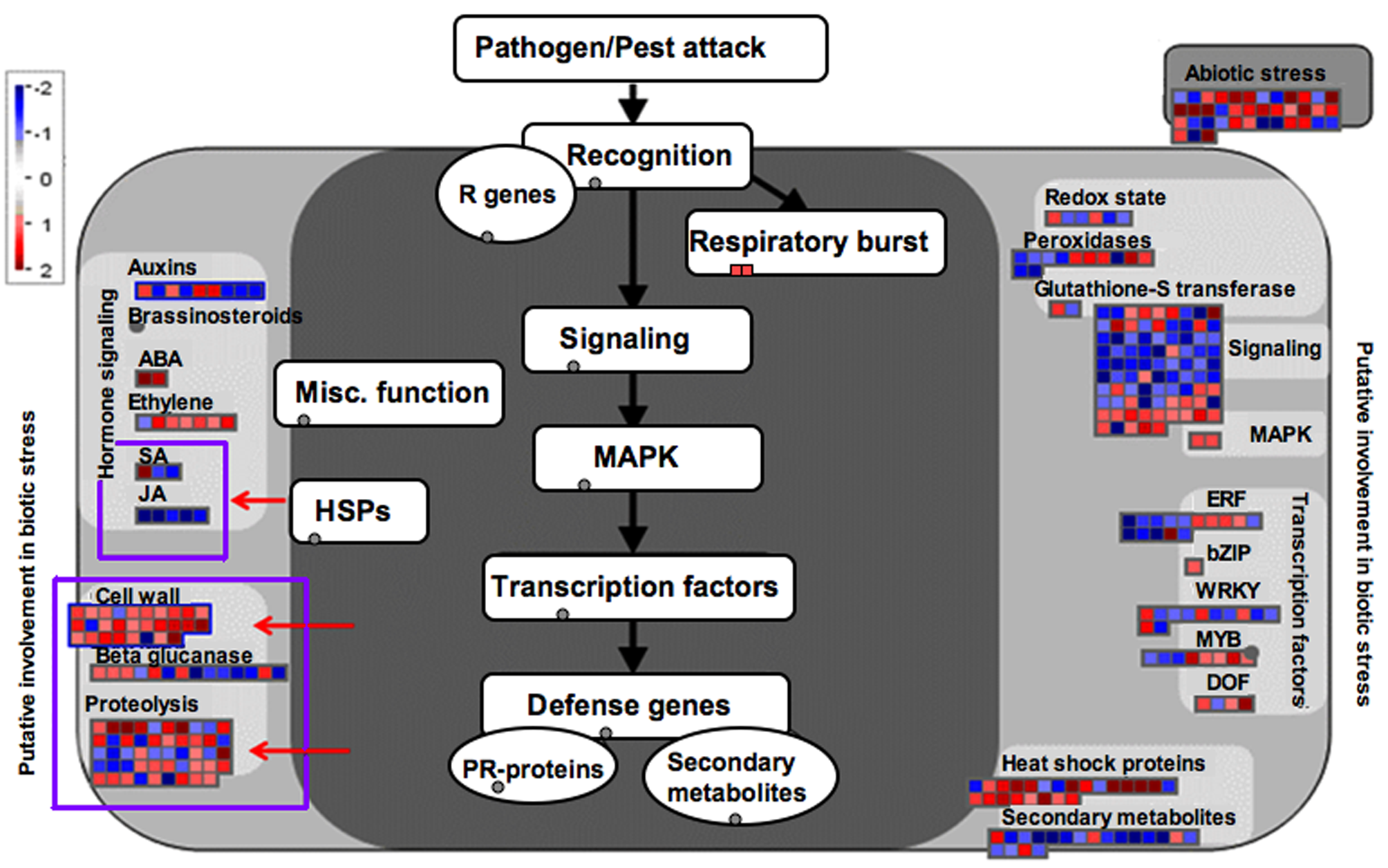

Supplement: Supplementary file 6 — Additional file 6:Figure S5. MapMan-based visualization of the differentially expressed genes involved in 'biotic stresses' in the microarray analysis of the rice variety Kavya after infestation with GMB4M. Functional subBINs (small squares) shown in red or blue indicate their up-regulation or down-regulation, respectively. Differentially regulated genes are marked with red arrows. Purple rectangles represent differentially expressed genes involved in hormone signaling; proteolysis; cell wall synthesis, modification and degradation. Grey circles indicate the genes unchanged or changed by less than 2-fold. Colour key represents log2 scale. (TIFF 7 MB) [file 12284_2011_8_MOESM6_ESM.TIFF]

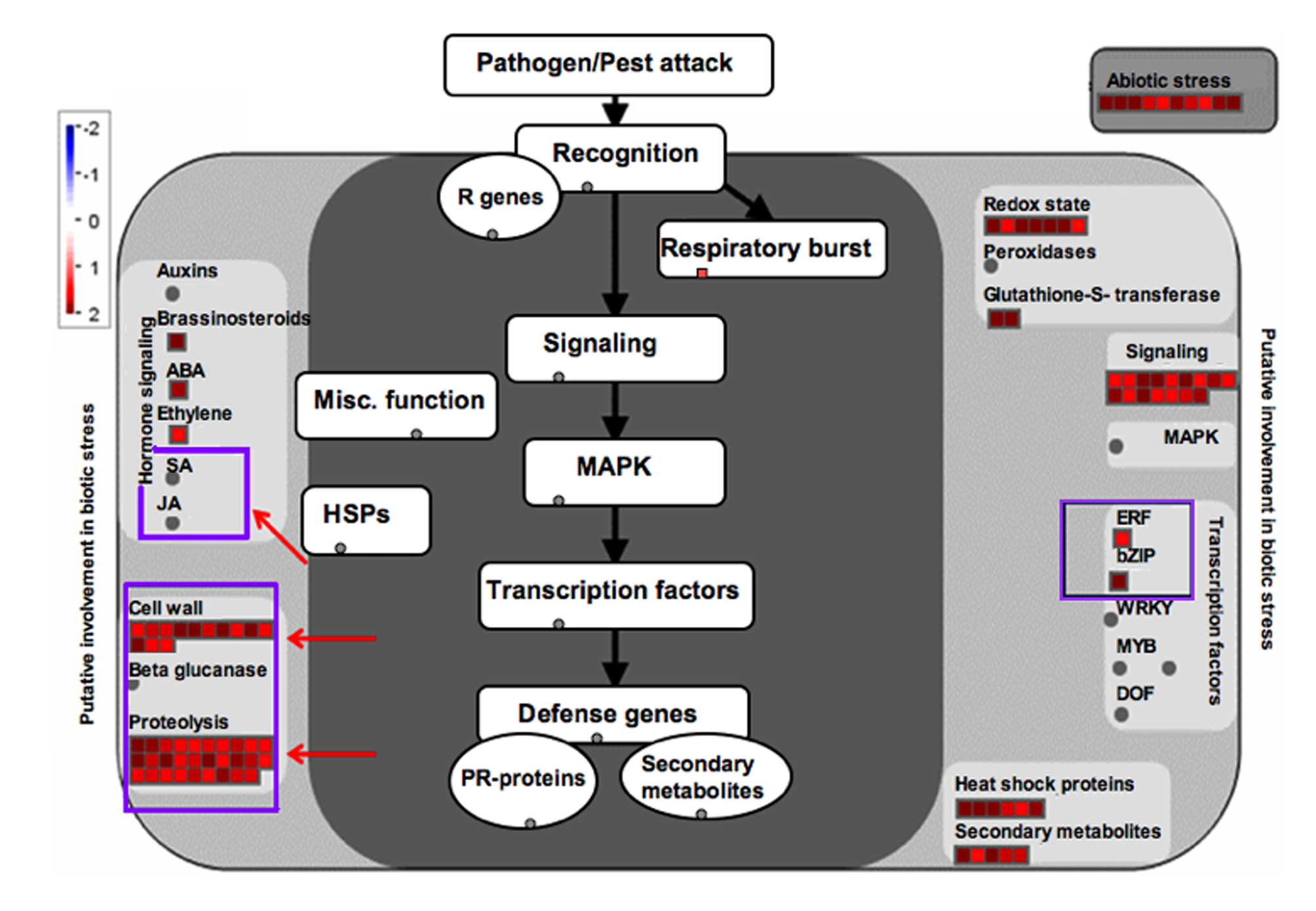

Supplement: Supplementary file 7 — Additional file 7:Figure S6. MapMan-based visualization of the differentially expressed ESTs involved in 'biotic stresses' in the SSH cDNA library of the rice variety TN1 after infestation with GMB4. Functional subBINs (small squares) shown in red indicate their up-regulation. Differentially regulated genes are marked with red arrows. Purple rectangles represent differentially expressed genes involved in hormone signaling; cell wall synthesis, modification and degradation; proteolysis and transcription factors. Grey circles indicate the genes unchanged or changed by less than 2-fold. Colour key represents log2 scale. (TIFF 8 MB) [file 12284_2011_8_MOESM7_ESM.TIFF]

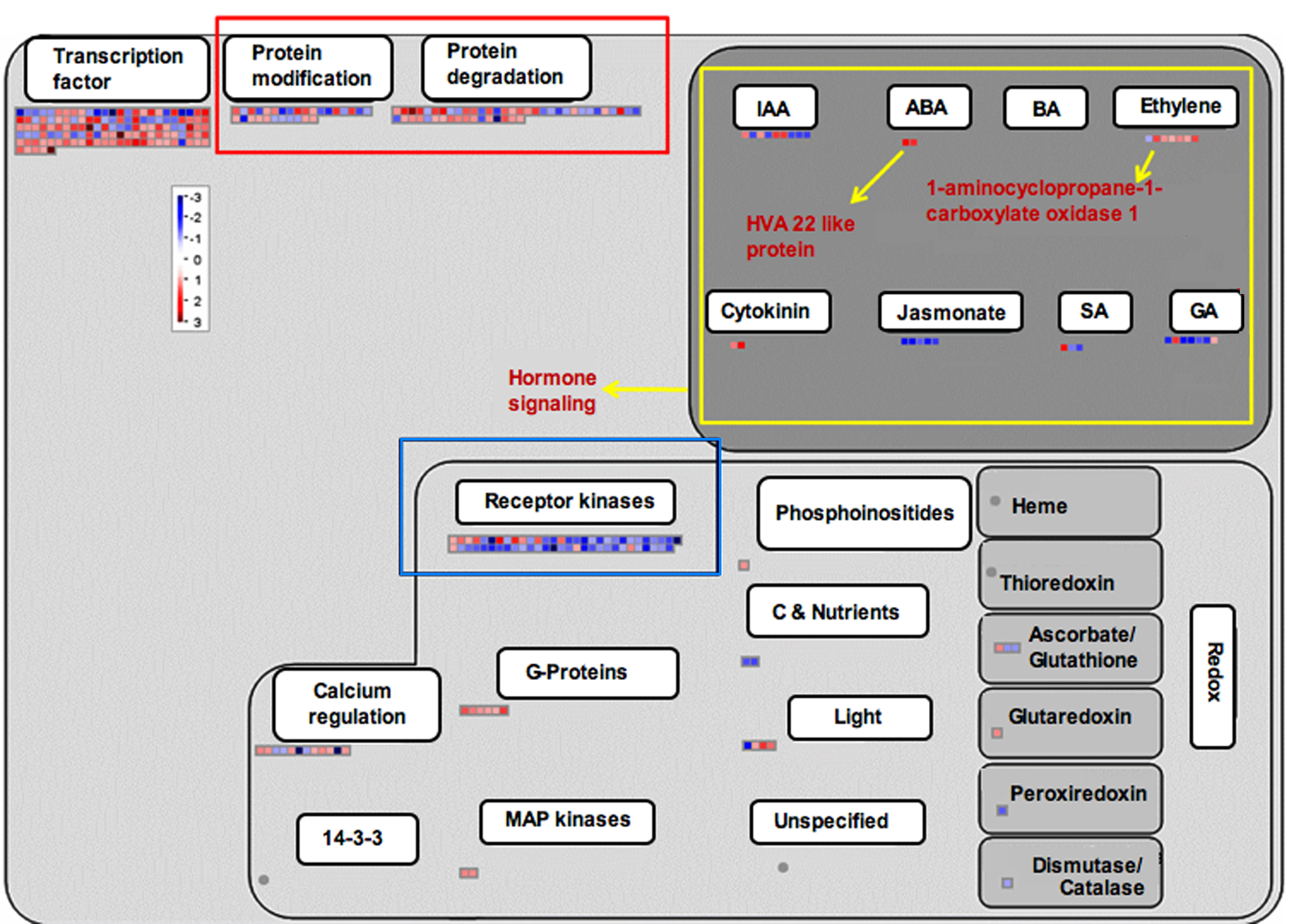

Supplement: Supplementary file 8 — Authors’ original file for figure 1 [file 12284_2011_8_MOESM8_ESM.tiff]

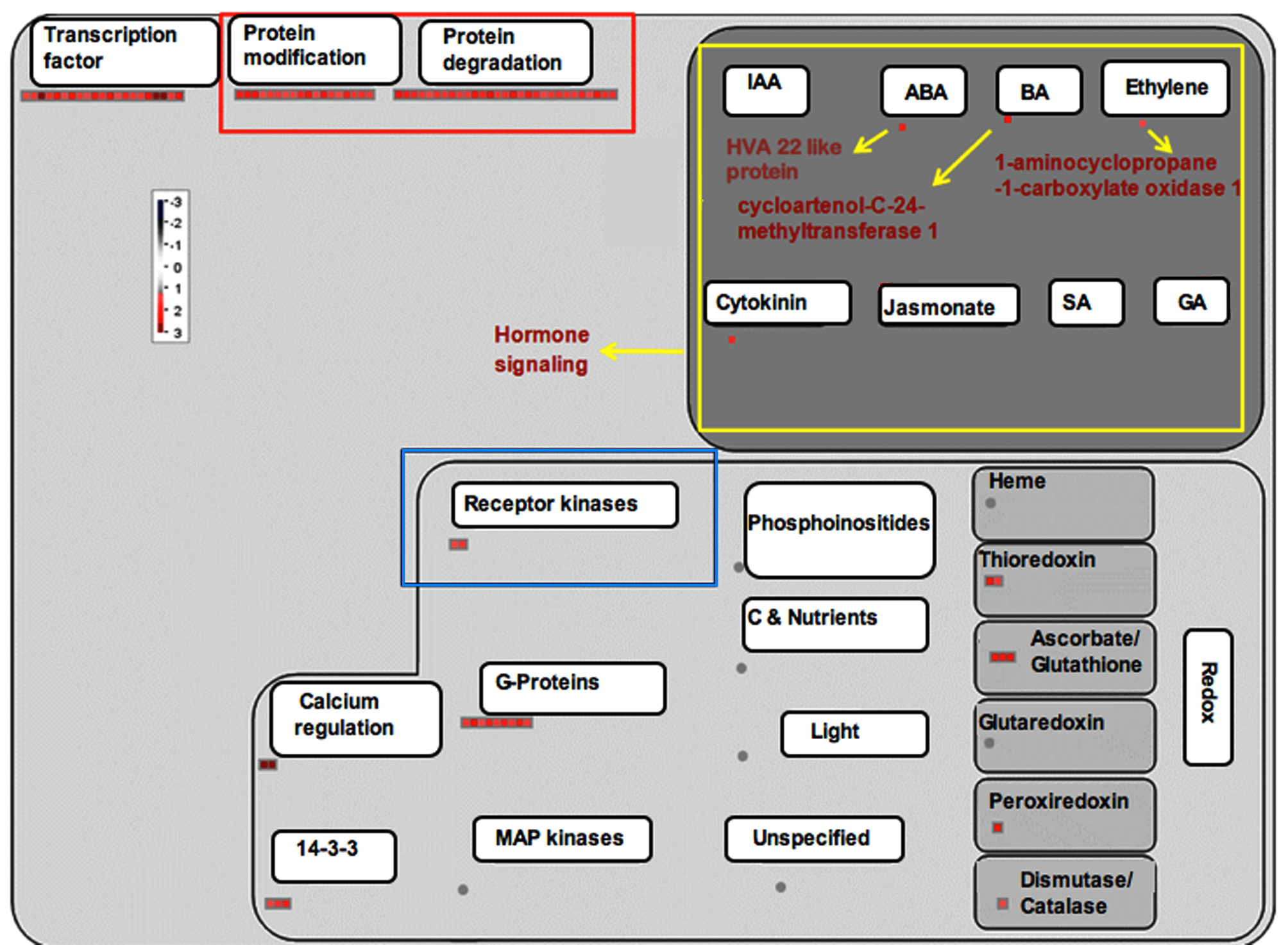

Supplement: Supplementary file 9 — Authors’ original file for figure 2 [file 12284_2011_8_MOESM9_ESM.tiff]

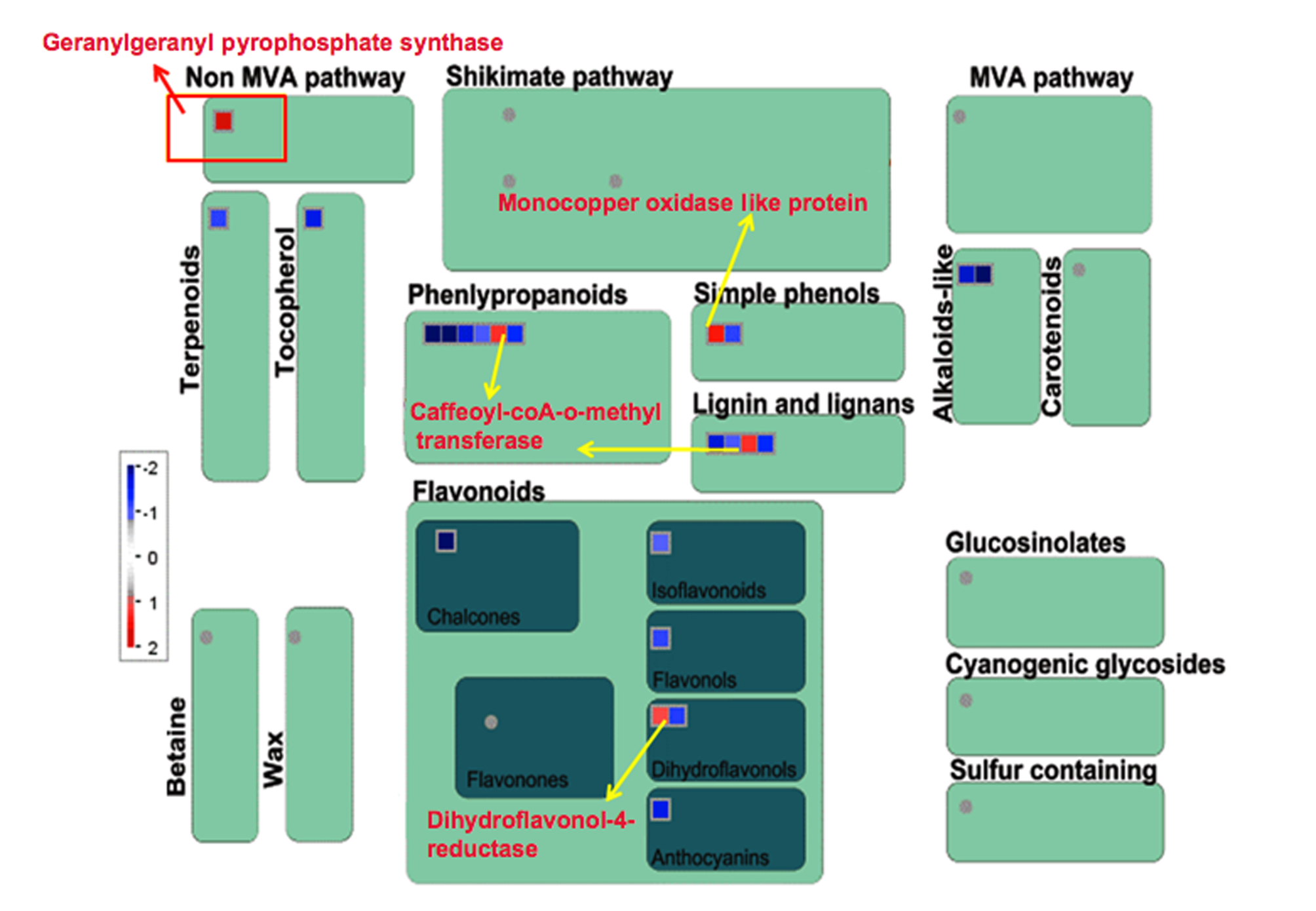

Supplement: Supplementary file 10 — Authors’ original file for figure 3 [file 12284_2011_8_MOESM10_ESM.tiff]

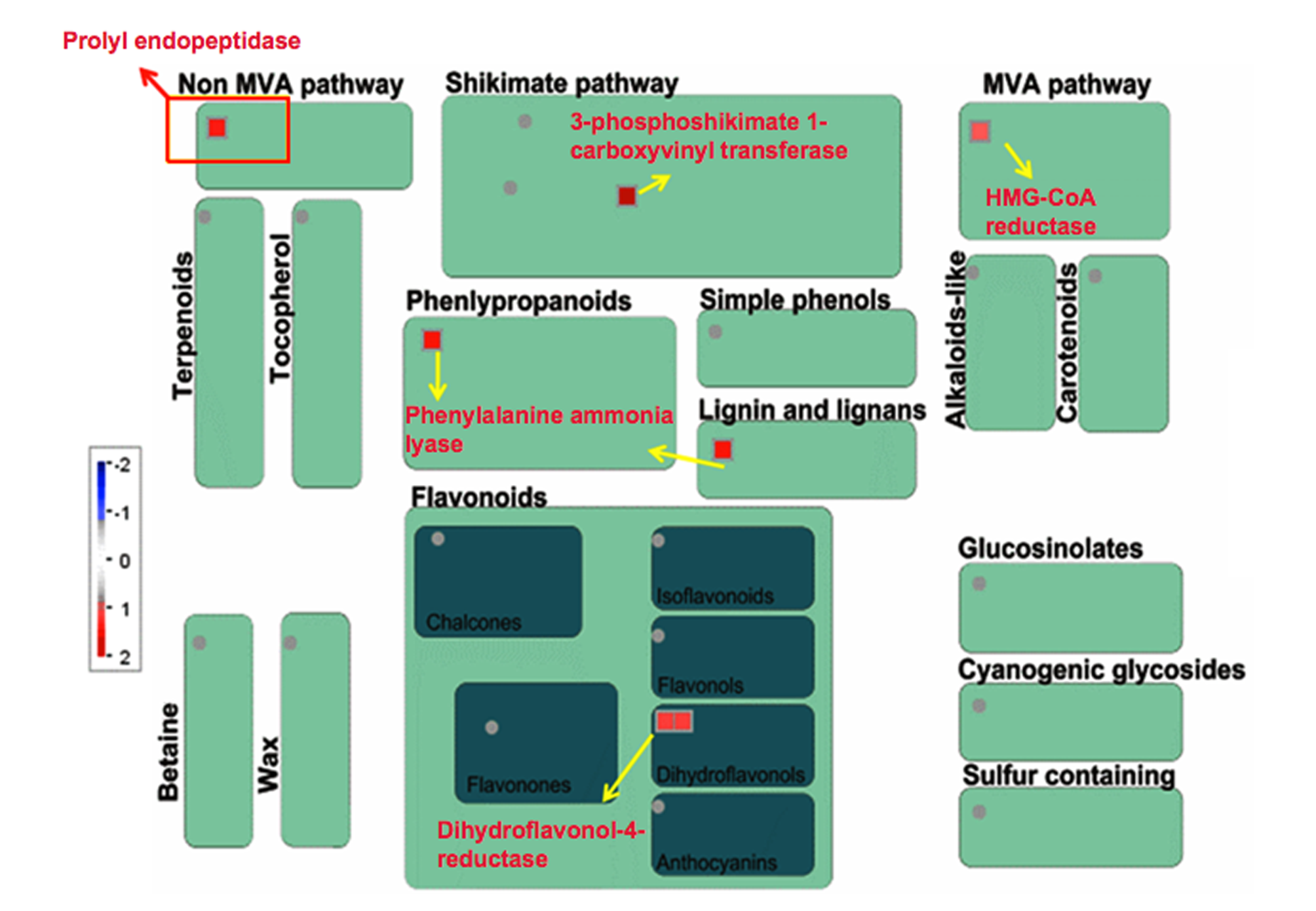

Supplement: Supplementary file 11 — Authors’ original file for figure 4 [file 12284_2011_8_MOESM11_ESM.tiff]

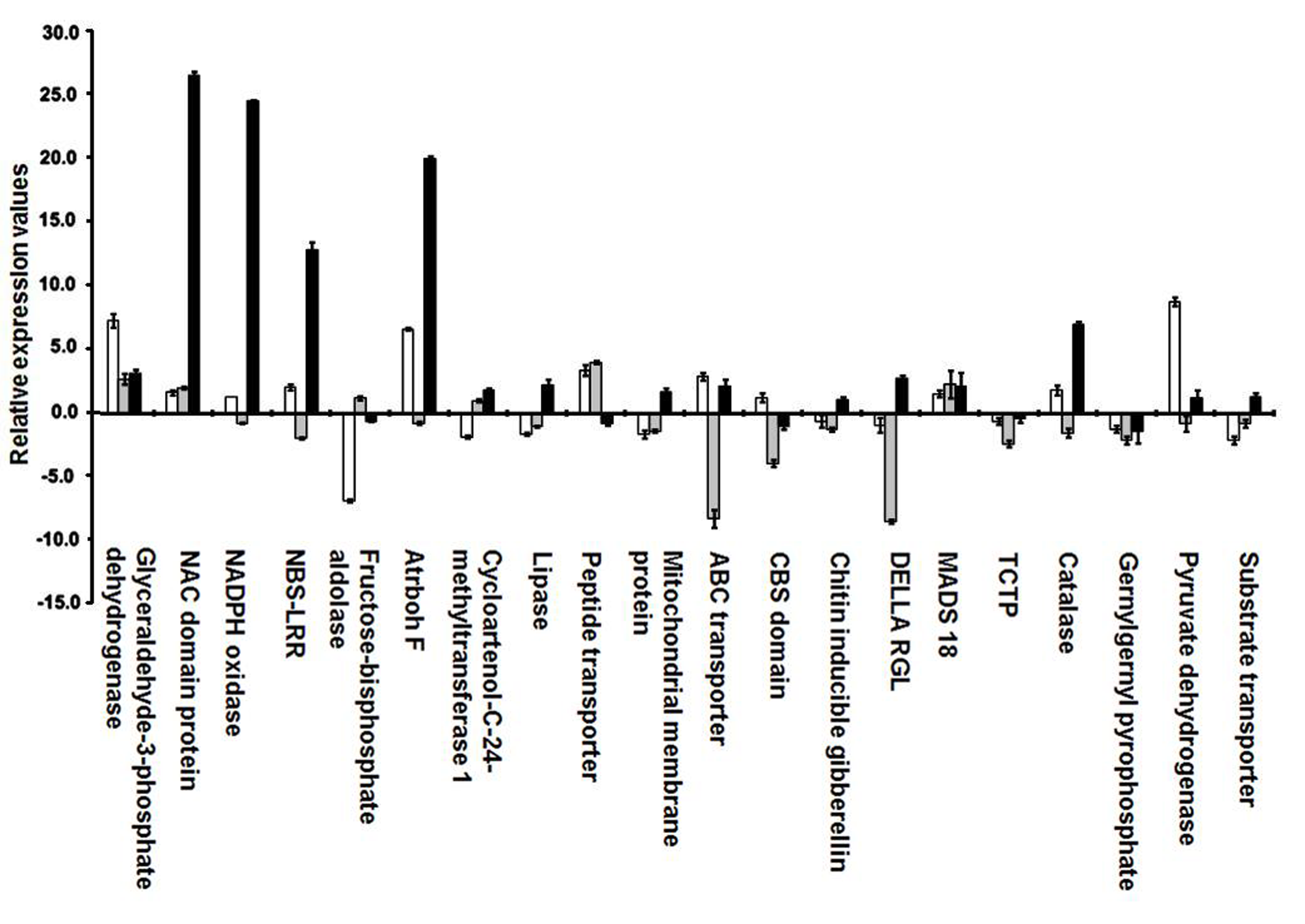

Supplement: Supplementary file 12 — Authors’ original file for figure 5 [file 12284_2011_8_MOESM12_ESM.tiff]

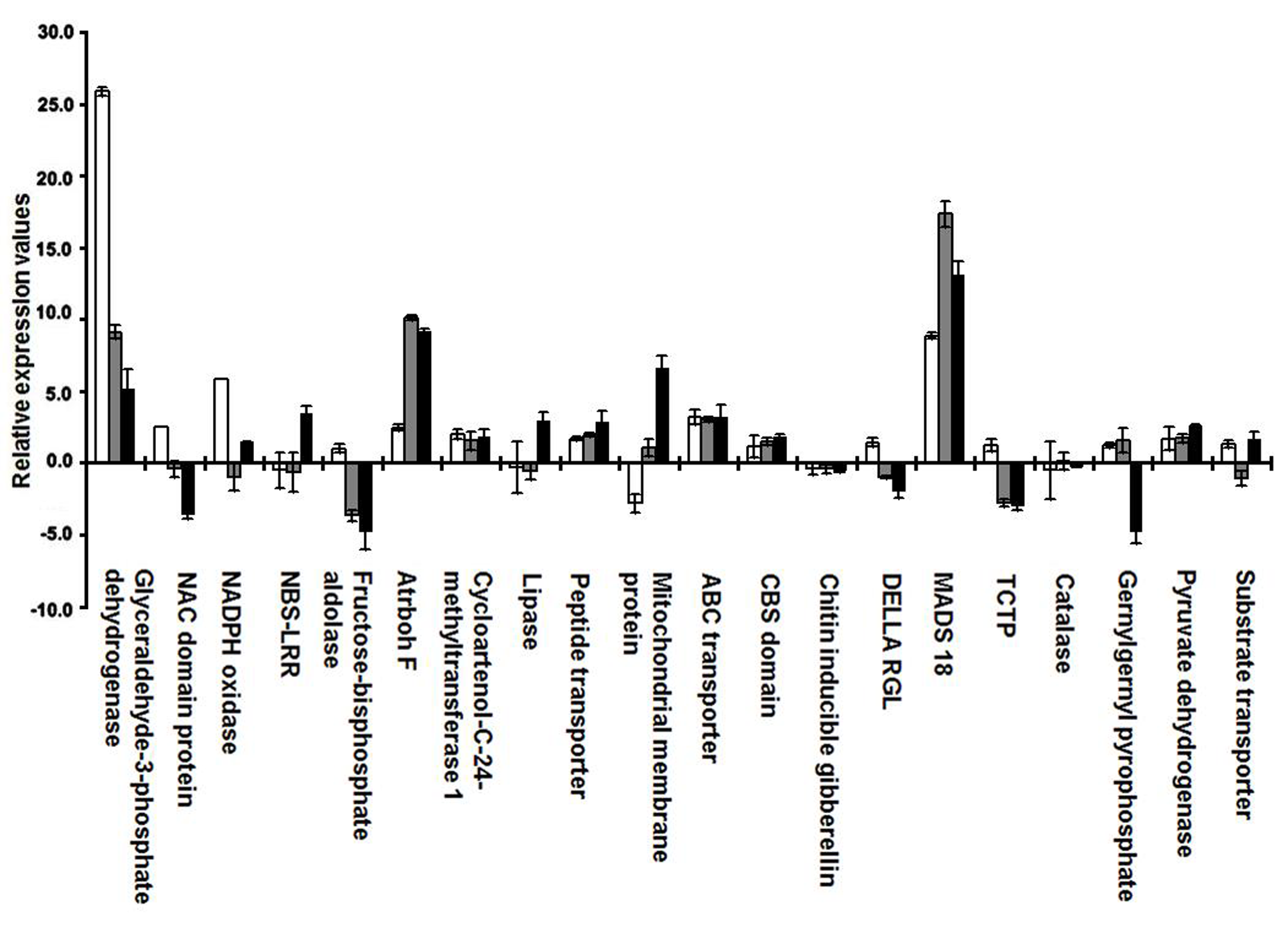

Supplement: Supplementary file 13 — Authors’ original file for figure 6 [file 12284_2011_8_MOESM13_ESM.tiff]
